# Benchmarking Lysosome Enrichment Methods: A Guide for Research and Clinical Translation

**DOI:** 10.1021/acs.analchem.5c05792

**Published:** 2026-02-03

**Authors:** Anniek L. de Jager, Sara Kassem, Louis Alesha, Brigitta A.E. Naber, Inge F. de Laat, Bas de Mooij, Kyra van der Pan, Erik Bos, Roman I. Koning, Jacques J.M. van Dongen, Cristina Teodosio, Paula Díez

**Affiliations:** † Department of Immunology, 16779 Leiden University Medical Center (LUMC), Leiden 2333ZA, The Netherlands; ‡ Electron Microscopy Facility, Department of Cell and Chemical Biology, 4501 Leiden University Medical Center (LUMC), Leiden 2333ZA, The Netherlands; § Department of Medicine, Translational and Clinical Research Program, Cancer Research Center, (IBMCC, University of Salamanca - CSIC), Cytometry Service, NUCLEUS, University of Salamanca and Institute of Biomedical Research of Salamanca (IBSAL), Salamanca 37007, Spain; ∥ Department of Functional Biology (Immunology Area), Faculty of Medicine and Health Sciences, University of Oviedo (UNIOVI), Oviedo 33006, Spain

## Abstract

Lysosomes, essential organelles involved in diverse cellular processes, are increasingly recognized as central players in the pathogenesis of numerous diseases. Due to their low abundance in whole-cell extracts, enrichment strategies are required for downstream analyses such as proteomics. Despite the availability of various lysosome isolation methods, including density gradient-based separation, filter-based approaches, magnetic bead-based isolation, and subcellular fractionation, a systematic, multimodal comparison of their performance is lacking. Here, four widely used lysosome enrichment techniques are benchmarked using the THP-1 monocytic cell line as a model. Each method has been evaluated for yield, purity, membrane integrity, reproducibility, scalability, and cross-contamination, employing nanoparticle tracking analysis, electron microscopy, flow cytometry, Western blotting, and mass spectrometry-based proteomics. Data reveal substantial differences: gradient-based and bead-based methods provide the highest lysosomal enrichment and proteomic purity, whereas the subcellular fractionation approach yields greater numbers of lysosomes but with increased variability and contamination. Finally, the filter-based method enables rapid processing, but mainly nonintact lysosomes are obtained with significant cross-contamination. These findings provide practical guidance for selecting the appropriate lysosome enrichment strategy, tailored to specific research or clinical objectives. The results also emphasize the need for rigorous validation to ensure the robustness of lysosomal studies in both basic and clinical research settings.

## Graphical abstract

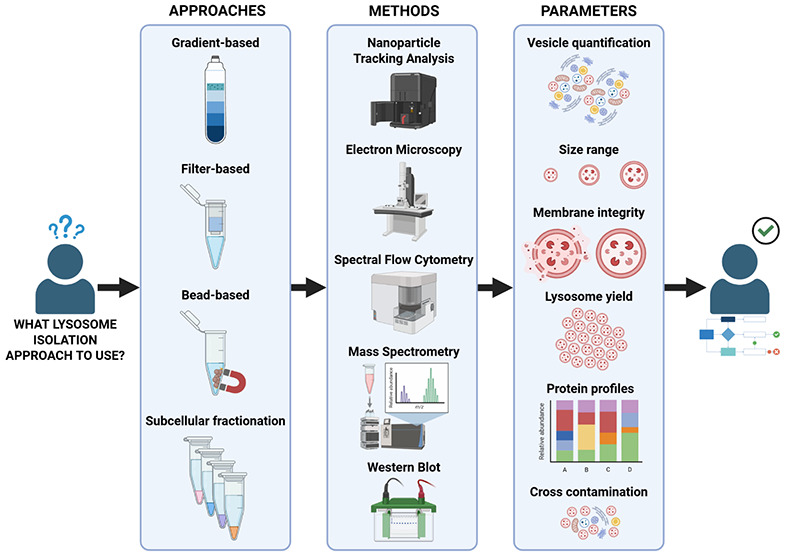

Since their discovery by De Duve et al.^1^ in 1955, lysosomes have emerged as dynamic organelles critical to cellular homeostasis, regulating processes from nutrient sensing to plasma membrane repair.^2–5^ These acidic, enzyme-packed vesicles^6,7^ are now acknowledged as central players in pathologies far beyond their classical association with lysosomal storage disorders (LSDs). Dysfunctional lysosomes are involved in neurodegenerative diseases (e.g., Alzheimer’s, Parkinson’s),^8–10^ cancer metastasis,^11–13^ and cardiovascular disorders.^14,15^ At the same time, lysosomal proteins like GBA1 and LAMP2 serve as biomarkers and therapeutic targets.^16–19^ Despite their clinical significance, a major challenge in lysosomal research persists since lysosomes constitute less than 5% of cellular volume,^20,21^ requiring precise enrichment for accurate analysis in diagnostic and therapeutic development.^22,23^

Current lysosome isolation methods, including density gradient-based separation,^24–28^ filter-based size exclusion approaches,^29^ magnetic particle phagocytosis,^30–33^ subcellular fractionation (SCF),^34^ and immunoprecipitation strategies,^35,36^ vary widely in yield, purity, structural integrity, and practicality. For instance, density gradient-based methods, which often use Percoll^37^ or sucrose,^38^ while well-established, user-friendly, and cost-effective, coisolate organelles with similar densities, such as endosomes, peroxisomes, and mitochondria,^39,40^ and can be time-consuming, potentially affecting lysosomal protein integrity. Conversely, filter-based size exclusion methods isolate lysosomes based on their size,^29^ offering shorter processing times and straightforward protocols. Nevertheless, these approaches may result in cross-contamination, as various cellular vesicles share similar sizes.^20,21^ Magnetic bead-based methods (e.g., superparamagnetic iron oxide nanoparticles, SPION) involve the endocytic uptake of magnetic beads,^35,41^ offering specificity but altering lysosomal physiology through forced phagocytosis, which hampers functional assays, such as in cancer drug screening.^42,43^ Finally, SCF, which employs sequential lysis steps to enrich specific organelles, including lysosomes,^34,44^ is a rapid, easy-to-use, and cost-effective method; however, significant cross-contamination is linked to this method.^34,44^ Such trade-offs are rarely addressed in single-modality studies, leaving clinicians and researchers without clear guidance for protocol selection. This gap is particularly critical in translational contexts, where diagnostic laboratories that prioritize high-purity lysosomes for biomarker discovery require protocols different from those of therapeutic teams that need lysosomes for drug efficacy testing.

Previous comparative studies, such as the proteomic analysis by Singh et al.,^35^ have provided valuable insights but primarily focused on yield and purity assessments via proteomic markers, overlooking several key parameters with potentially relevant impacts in translational and clinical applications. Critically, these studies often omit functional and practical metrics: for instance, they typically do not evaluate lysosomal membrane integrity, which is vital for enzyme activity and therapeutic testing, nor do they consider the scalability and feasibility of protocols for contexts where only limited patient samples are available or the potential presence of isolation artifacts. This omission of practical, multimodal assessment represents a critical gap that prevents researchers from selecting a protocol optimized for specific clinical goals.

Our study seeks to address these limitations by employing a multimodal framework that evaluates four widely used lysosome isolation methods across several metrics that are critical for clinical applications. We systematically assessed the yield and reproducibility of each method, factors that are essential for biobanking and high-throughput diagnostic workflows. We also examined organelle purity, crucial for avoiding false positives in proteomic biomarker screenings, together with lysosomal membrane integrity, an important consideration for functional studies and drug testing. Finally, we considered the scalability of each method, ensuring that our findings are relevant for studies involving rare or precious patient-derived cells together with the cost and processing time of each technique. Thus, by integrating nanoparticle tracking analysis (NTA), spectral flow cytometry, transmission electron microscopy (TEM), and mass spectrometry (MS)-based proteomics, our study revealed distinct advantages for each method depending on the intended application. With this comprehensive benchmark, we bridged a critical gap between fundamental lysosome biology and clinical implementation, guiding researchers and clinicians in the selection of the most appropriate isolation strategies tailored to their diagnostic or therapeutic goals.

## Experimental Section

### Cell Culture

The acute monocytic leukemia THP-1 cell line (ACC 16, DSMZ, Braunschweig, Germany) was cultured in RPMI 1640 (VWR International, Radnor, PA/USA) supplemented with 10% v/v heat-inactivated fetal calf serum (hiFCS, Bodinco, Alkmaar, The Netherlands), 200 μg/mL of streptomycin (Gibco, Gaithersburg, MD/USA), 200 U/mL of penicillin (Gibco), and 1% v/v GlutaMAX Supplement (Gibco).

### Lysosome Enrichment Approaches

In all cases, 4 × 10^7^ THP-1 cells were used as starting material for the purification of lysosomes according to the methods detailed next.

### Gradient-Based Isolation Methodology

Gradient-based lysosome isolation was performed using the Lysosome Enrichment Kit for Tissues and Cultured Cells (Thermo Fisher, Waltham, MA/USA) according to the manufacturer’s instructions. Briefly, cell pellets containing 4 × 10^7^ cells were resuspended in 800 μL of ice-cold reagent A and lysed on ice using a Branson Digital Sonifier 250 (Emerson Electric, St. Louis, MO/USA), with 15 pulses of 3 s at 15% amplitude. After lysis, 800 μL of ice-cold reagent B was added, and cellular debris was removed by centrifugation at 500 *g* for 10 min. The supernatants were then diluted to a total volume of 2.4 mL with Gradient Dilution Buffer, after which 800 μL of Cell Separation Media was added. The sample was then carefully layered onto a discontinuous five-layer density gradient and centrifuged in an Optima XE-90 ultracentrifuge (Beckman Coulter, Brea, CA/USA) at 145,000 *g* for 2 h at 4 °C using an SW-40-Ti swinging-bucket rotor. Following centrifugation, the lysosome-enriched top target layer was collected, diluted 3-fold with sterile, filtered phosphate-buffered saline (PBS, pH 7.4; Fresenius Kabi, Frankfurt am Main, Germany), and centrifuged at 18,000 *g* for 30 min at 4 °C. The resulting pellet was resuspended in 1 mL Gradient Dilution Buffer and centrifuged again at 18,000 *g* for 30 min to remove residual Cell Separation Media. The final pellet, containing the lysosome-enriched fraction, was kept on ice for downstream applications.

### Filter-Based Methodology

Filter-based lysosome isolation was performed using the Minute Lysosome Isolation Kit for Mammalian Cells/Tissues (Invent Biotechnologies, Plymouth, MN/USA) employing spin columns, following the manufacturers’ instructions. Briefly, cell pellets containing 4 × 10^7^ cells were resuspended in 500 μL ice-cold buffer A and vortexed for 30 s to lyse the cells, followed by centrifugation at 16,000 *g* for 10 s employing a filter cartridge. The filtered suspension was then centrifuged at 2,000 *g* for 3 min to remove the nuclei, resuspended in PBS, and centrifuged for 15 min at 11,000 *g* to eliminate mitochondria. The nuclei- and mitochondria-depleted supernatant was further centrifuged at 16,000 *g* for 30 min, and the obtained pellet was resuspended in 200 μL ice-cold buffer A (by pipetting up and down 100 times), followed by a vigorous vortex for 20 s and centrifugation at 2,000 *g* for 4 min. The supernatant was then mixed with 100 μL of ice-cold buffer B (2:1 ratio), incubated on ice for 30 min, and centrifuged at 11,000 *g* for 10 min to obtain a lysosome-enriched pellet.

### Bead-Based Methodology

The bead-based strategy is based on the magnetic isolation of phagolysosomes after phagocytosis of DexoMAG 40 magnetic nanoparticles (Liquids Research Limited, Bangor, UK), according to the manufacturer’s protocol. Briefly, a total of 100 mL with 4 × 10^5^ cells/mL were incubated with complete culture medium supplemented with 10% v/v DexoMAG 40 magnetic nanoparticles and 10 mM HEPES (Sigma-Aldrich, St. Louis, MO/USA) for 20 h, followed by a pulse phase of 21 h in nanoparticle-free culture medium, both at 37 °C in a 5% CO_2_-containing atmosphere. Subsequently, cells were centrifuged, resuspended in hypotonic buffer A (15 mM KCl, 1.5 mM MgAc, 1 mM DTT, 10 mM HEPES, 1 μL of protease cocktail inhibitor, total volume 50 mL of distilled H_2_O [dH_2_O]), homogenized using a 7 mL Dounce tissue grinder (Sigma-Aldrich) for 30 strokes, and passed 8× through a 23-gauge needle. Next, the sample was incubated in buffer B (220 mM HEPES pH 7.2, 0.1 mM sucrose, 375 mM KCl, 22.5 mM MgAc, 1 mM DTT, 50 μL of DNase I, and total volume 5 mL of dH_2_O) on ice for 5 min, followed by centrifugation at 200 *g* for 10 min to remove cellular debris, mainly being remnants of cell membranes. The supernatant was then passed through a 0.5% bovine serum albumin (BSA, Sigma-Aldrich)-equilibrated LS MACS column (Miltenyi Biotec, Bergisch Gladbach, Germany) placed in a MidiMACS separator to bind the magnetic nanoparticle-loaded vesicles. After washing the column with subsequent 0.1 mM DNase I (Sigma-Aldrich) and 0.1 mM sucrose (Sigma-Aldrich) solutions, the (phago)­lysosomal fraction was eluted from the column and used for further analysis. If a pellet was needed for further analysis, the enriched sample was pelleted by centrifugation at 18,000 *g* for 30 min.

### Subcellular Fractionation (SCF) Methodology

For this approach, we applied a method described by Díez et al.^34^ for the separation of cellular compartments using detergents. In short, 40 million cells were lysed in a hypotonic buffer (30 mM HEPES, 15 mM KCl, 2 mM MgCl_2_, 20% glycerol, 1% phosphatase/protease inhibitor cocktails, 1 mM dithiothreitol (DTT), 1 mM phenylmethylsulfonyl fluoride (PMSF), and 1 mM EDTA, all from Sigma-Aldrich) containing 0.015% (w/v) digitonin (Sigma-Aldrich) and 1% (v/v) protease inhibitor cocktail (Sigma-Aldrich). The cell suspension was incubated on ice with gentle rotation for 30 min to allow for selective permeabilization. Following incubation, the lysate was centrifuged at 1,500 *g* for 5 min. The resulting pellet, enriched for the lysosomal fraction, was resuspended in PBS for downstream analyses.

### Strategies for the Assessment of Lysosome Enrichment Approaches

#### Nanoparticle Tracking Analysis

Freshly isolated lysosome samples were analyzed by nanoparticle tracking analysis (NTA) employing the NanoSight NS300 (Malvern Panalytical, Malvern, United Kingdom), equipped with the NanoSight NTA software v3.2 (Malvern Panalytical), to assess particle concentration and size distribution. Pelleted samples were resuspended and diluted in PBS to obtain the ideal particle per frame value and injected at a flow rate of 50 psi using a syringe pump. The camera level was set to 14, and the detection threshold was set to 10 for all samples.

#### Transmission Electron Microscopy

Pelleted isolated lysosomes were attached to a Formvar and carbon-coated copper electron microscopy grid that was previously glow discharged in air at 20 mA for 1 min. The grid with the attached lysosomes was rinsed on droplets of PBS, followed by rinsing on water. The grids were then incubated for 5 min on ice in 2% methylcellulose (Sigma-Aldrich) and 0.6% uranyl acetate (EMS, Hatfield, PA/USA) in water. Excess of the staining solution was removed by blotting on a filter paper. The grids were air-dried and imaged at 120 kV on a Tecnai 12 electron microscope (Thermo Fisher) equipped with a 4k × 4k Eagle camera (Thermo Fisher).

#### Spectral Flow Cytometry

Lysosome fractions were resuspended in PBS and stained with 1 μM LysoTracker Deep Red (Thermo Fisher; for the staining of acidic compartments) and 10 μM Calcein AM (C1430, Thermo Fisher; for the identification of membrane intactness) (final staining volume: 100 μL) for 20 min at 37 °C. After incubation, samples were placed on ice and diluted with PBS up to 150 μL, followed by direct measurement on a 5L Cytek Aurora (Cytek Biosciences, Fremont, CA/USA) spectral flow cytometer. If needed, samples were further diluted to maintain a flow rate of <10,000 events/sec during acquisition.

#### Flow Cytometer Setup and Data Analysis

To optimize the lysosome measurement, several steps were taken to minimize background and ensure consistency across measurements. First, to reduce background noise, the standard 0.2 μm sheath filter was replaced with a 0.04 μm version, and the sheath fluid was substituted with filtered PBS. Additionally, a long cleaning procedure was performed before measurements to maintain optimal instrument cleanliness.

Measurement was performed by using a side scatter area (SSC-A) threshold of 700. To reduce variation between measurements, 200 nm Fluorescent Sky Blue Particles (Spherotech, Lake Forest, IL/USA) were used to standardize the forward scatter area (FSC-A) and SSC-A values. The FSC and SSC detector gains were adjusted to target values of 5,300 and 9,298, respectively. Unstained and single-stained samples were used to assess the potential background signal.

Analysis was performed employing virtual filters (Calcein B1–B2; LysoTracker Deep Red, R2–R3) in the SpectroFlo software version 3.0.3 (Cytek Biosciences) to increase discrimination of weak fluorescence from negative events. The Infinicyt Flow Cytometry Software version 2.0.5.d (Cytognos, Salamanca, Spain) was used for data analysis.

#### Protein Extraction and Quantification

Lysosomal fractions were lysed as described by van der Pan et al.^45^ In short, pelleted fractions were lysed in 9 M urea (Thermo Fisher), 20 mM HEPES pH 8.5, 1 mM sodium orthovanadate, 2.5 mM sodium pyrophosphate, 1 mM β-glycerophosphate, 1% phosphatase/protease inhibitor cocktails, and 10 μg/mL DNase I (all from Sigma-Aldrich), followed by 3 cycles of 5 s at 15 W of sonication in an ice–water bath. Samples were then centrifuged at 21,000 *g* for 15 min, and supernatants containing the extracted proteins were transferred to a Protein LoBind tube (Eppendorf, Hamburg, Germany) to decrease protein losses and stored at −80 °C until further processing. Extracted proteins were quantified using the Qubit Protein Assay Kit (Thermo Fisher) on a Qubit 3.0 Fluorometer (Thermo Fisher) following the manufacturer’s protocol.

#### Western Blot

Extracted proteins were subsequently heated at 99 °C for 5 min in sample buffer (10% (v/v) glycerol, 0.05 M Tris-HCl pH 6.8, 2% (w/v) SDS, bromophenol blue), loaded onto a Novex 12% Tris-glycine mini gel (Thermo Fisher), and run at 200 V. For detection of low molecular weight proteins (LMWPs), samples were heated at 85 °C for 2 min in Novex Tricine SDS Sample Buffer (Thermo Fisher) and together with the Precision Plus Protein WesternC Blotting Standard (Bio-Rad Laboratories, Hercules, CA/USA) run in a Novex 16% Tricine gel (Thermo Fisher) at 125 V. After electrophoresis, proteins were transferred to polyvinylidene difluoride (PVDF) membranes (iBlot 2 Transfer Stacks, Thermo Fisher) employing the iBlot 2 Gel Transfer Device (Thermo Fisher; 6 or 8 min at 20 V for standard proteins and LMWP, respectively). Transfer assessment was performed using the Pierce Reversible Protein Stain Kit for PVDF Membranes (Thermo Fisher) following the manufacturer’s protocol. Membranes were blocked with iBind Flex Solution for 5 min and incubated with primary antibodies directed at diverse organelles to detect potential subcellular cross-contamination: peroxisome (ABCD3, 1G7G5, Proteintech, Rosemont, IL/USA; 1:500 dilution), endoplasmic reticulum (CLNX, CANX/1541, Thermo Fisher; 1:200 dilution), mitochondria (COX4, 4D11-B3-E8, Cell Signaling, Danvers, MA/USA; 1:1,500 dilution), lysosome (CTSB, D1C7Y, Cell Signaling; 1:100 dilution), endosomes (EEA1, G-4, Santa Cruz Biotechnology, Dallas, TX/USA; 1:100 dilution), and Golgi apparatus (GM130, 10H5L5, Thermo Fisher; 1:250 dilution). Next, membranes were incubated with a secondary antimouse horseradish peroxidase antibody (HRP, Agilent, Santa Clara, CA/USA; 1:1,500 dilution). All antibody incubations were performed using the iBind Flex Western Device (Thermo Fisher). Protein-antibody binding was detected by chemiluminescence with a SuperSignal West Pico PLUS Chemiluminescent Substrate (Thermo Fisher). Images were obtained using the ChemiDoc MP Imaging System (Bio-Rad Laboratories) and analyzed using Image Lab (version 6.1.0, Bio-Rad Laboratories).

#### Liquid Chromatography–Tandem Mass Spectrometry Analysis (LC–MS/MS)

For liquid chromatography–tandem mass spectrometry (LC–MS/MS) studies, a total of 15 μg of proteins were reduced with 10 mM DTT at 45 °C for 30 min, followed by alkylation with 40 mM 2-iodoacetamide (IAA, Sigma-Aldrich) at RT for 30 min. This last reaction was quenched with 20 mM DTT, and protein digestion and peptide recovery were performed using hydrophilic and hydrophobic beads (Sera-Mag Carboxylate-Modified Magnetic Particles, Cytiva, Marlborough, MA/USA) according to the Single-Pot Solid-Phase-enhanced Sample Preparation (SP3) method.^46,47^ In short, samples were incubated twice with beads (1:10 (w/w) protein:bead ratio) in 70% acetonitrile (ACN) for 18 min at RT. After incubation, bead-bound proteins were retained by using a DynaMag-PCR Magnet (Thermo Fisher), and contaminants were washed away three times with 80% ethanol (Sigma-Aldrich). Proteins were digested using a Trypsin/LysC mix (1:25 (w/w) enzyme:protein ratio, Promega, Madison, WI/USA) in 50 mM ammonium bicarbonate at RT overnight. Beads were then washed twice with ACN, after which peptides were eluted in 2% (v/v) dimethyl sulfoxide (DMSO, Thermo Fisher), lyophilized in a freeze-dryer, and stored at −20 °C. Samples were then tagged using the tandem mass tag (TMT) labeling kit (TMTpro 16plex tags, Thermo Fisher) by resuspending the lyophilized peptides in 40 mM HEPES pH 8.4 and labeling with the TMT tags at a 1:5 (w/w) sample:tag ratio for 1 h at RT. The labeling reaction was quenched with 5% hydroxylamine. TMT-labeled lysosome samples were pooled, lyophilized in a freeze-dryer, and stored at −20 °C until LC–MS/MS analysis.

#### Mass Spectrometry Data Acquisition and Analysis

TMT-labeled peptides were dissolved in solvent A (water/formic acid (FA), 100/0.1 (v/v)) and analyzed by online C18 nano-HPLC MS/MS, coupling an UltiMate 3000 gradient HPLC system (Thermo Fisher) and an Orbitrap Exploris 480 mass spectrometer (Thermo Fisher). A total of 12 fractions were injected onto a precolumn (300 μm × 5 mm; PepMap100 C18 5 μm, Thermo Fisher) equilibrated with solvent A and eluted via a homemade analytical nano-HPLC column (50 cm × 75 μm; ReproSil-Pur C18-AQ 1.9 μm) using solvent B (80/20/0.1 (v/v/v) ACN/water/FA) in a gradient from 5 to 30% in 160 min. The nano-HPLC column was drawn to a tip of ∼5 μm and functioned as an electrospray needle. A Sonation PRSO-V2 column oven was used to maintain the analytical column temperature at 50 °C. The MS1 spectra were recorded in the Orbitrap at a mass-to-charge ratio (*m*/*z*) range of 350–1,600, a resolution of 120,000, a maximum injection time of 50 ms, and automatic gain control (AGC) at standard. Dynamic exclusion was applied after *n* = 1 with an exclusion duration of 45 s and a mass tolerance of 10 ppm. Charge states 2–5 were included. Precursors for MS2 were selected using a TopSpeed method of 3 s at a resolution of 45,000 and fragmented by high-energy collision-induced dissociation (HCD) at a normalized collision energy (NCE) of 36%, with an AGC at 200 and the maximum injection time set to auto. The isolation window for MS/MS was 1.2 Da.

Raw data files were converted to the peak list by using Proteome Discoverer v2.4 (Thermo Fisher), followed by protein identification employing the UniProtKB database (*Homo sapiens*, 20,596 entries) using Mascot v2.2.07 (Matrix Science Inc., Boston, MA/USA) with the following parameters: precursor and fragment mass tolerances of 10 ppm and 0.02 Da, respectively; TMT16plex on N-term and Lys as fixed modifications; oxidation on Met and acetylation of N-term as variable modifications; and full tryptic digestion (no P rule) with up to 2 missed cleavages. A false discovery rate (FDR) of 1% was set for peptide spectrum matches (PSMs), peptides, and proteins.

The MS data have been deposited via the PRIDE partner repository to the ProteomeXchange Consortium with the identifier PXD040003.

#### Statistical Analyses

For each continuous variable, the median ± range was calculated and plotted. Statistical significance (*p*-value < 0.05) between methods was determined using the nonparametric Kruskal–Wallis test and corrected for multiple comparisons using the original false discovery rate (FDR 5%) method of Benjamini and Hochberg. All statistical analyses were performed using Prism (version 9.3.1, GraphPad, San Diego, CA/USA).

## Results

### Quantification and Size Distribution of Isolated Vesicle Fractions

To assess the yield and size distribution of vesicles isolated by different methods, nanoparticle tracking analysis (NTA) was employed. The SCF procedure yielded the highest median number of vesicles per cell (2,356.3 ± 7,461.7; median ± range), followed by gradient-based (1,630.2 ± 1632.3), filter-based (228.2 ± 265.6), and bead-based isolation (27.8 ± 35.2). Both filter- and bead-based approaches resulted in significantly lower yields compared to SCF (*p*-value = 0.002 and *p*-value <0.0001, respectively), and the gradient-based method yielded significantly more vesicles than the bead-based approach (*p*-value = 0.002; Figure 1A).

**Figure 1:**
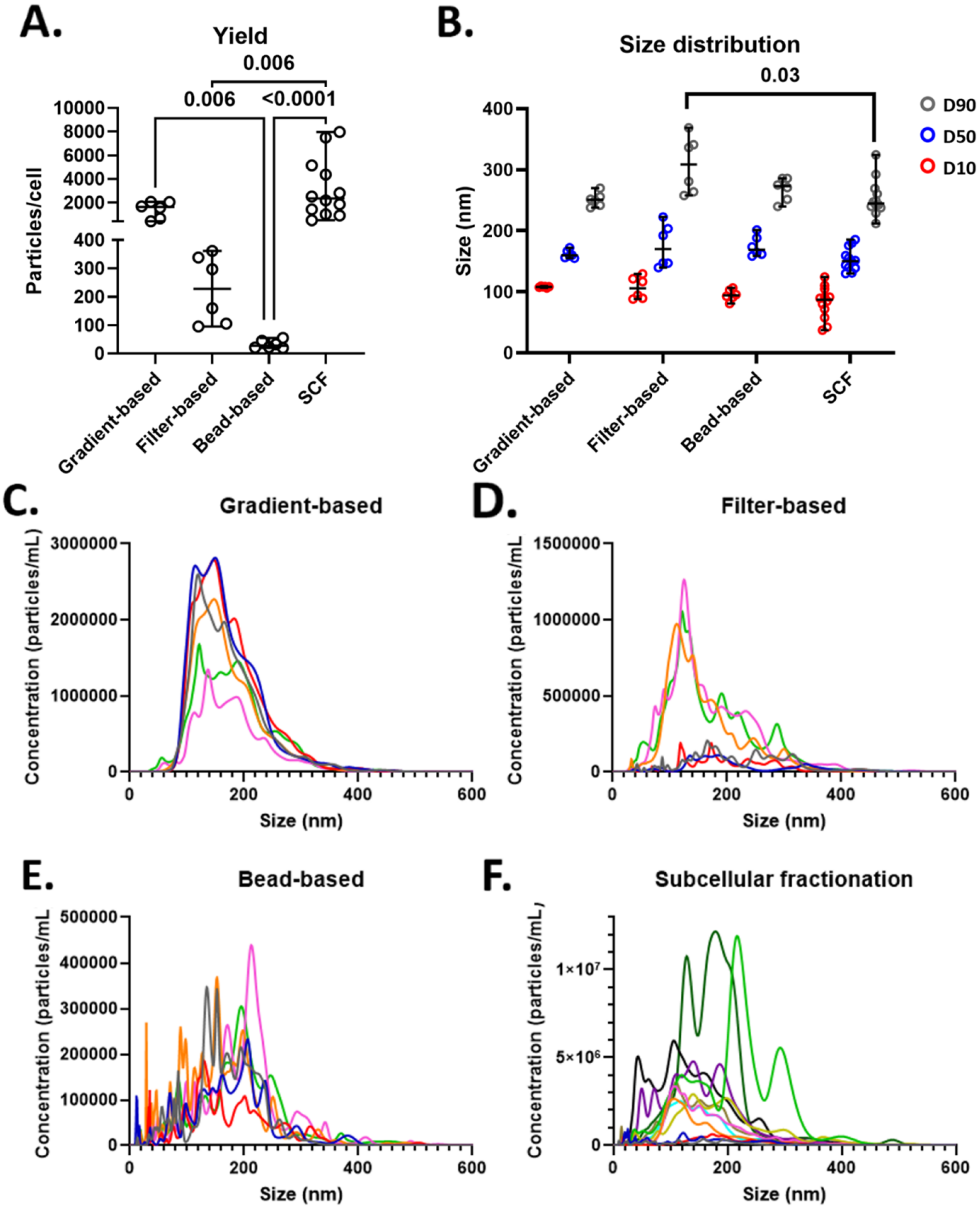
Quantification and size analyses of isolated vesicles. A) Quantification of isolated vesicles per cell, shown as median with range. Circles represent technical replicates. B) Size distribution of isolated vesicles (in nm), displaying average D10 (red), D50 (blue), and D90 (gray), depicted as median with range. Circles represent technical replicates. C–F) Particle distribution plots showing mean concentration at specific sizes; each line represents a technical replicate. For each method, six technical replicates are shown, except for SCF, which includes 12 replicates. SCF, subcellular fractionation. Statistical significance: *p* < 0.05 and FDR 5%. For statistical analysis, the nonparametric Kruskal–Wallis test was used and corrected for multiple comparisons using the method of Benjamini and Hochberg.

Median vesicle size (D50) was comparable across all methods: 159.5 ± 17.2 nm (gradient-based), 169.9 ± 82.7 nm (filter-based), 168.9 ± 42.4 nm (bead-based), and 150.0 ± 55.6 nm (SCF). Similarly, decile 10 (D10) values were consistent, ranging from 87.1 ± 87.3 nm (SCF) to 108.2 ± 3.1 nm (gradient-based). However, filter-based isolation led to higher size heterogeneity of the isolated particles, capturing significantly larger vesicles at the upper end (D90:308.5 ± 110.7 nm) compared to SCF (244.9 ± 112.6 nm, *p*-value = 0.005), while gradient- and bead-based methods showed intermediate D90 values (250.8 ± 31.7 nm and 273.0 ± 45.9 nm, respectively; Figure 1B). Notably, greater intra-assay variation in concentration and size distribution was observed with filter-based and SCF methods compared to the other approaches (Figure 1C–F).

### Morphology of the Isolated Fractions

Transmission electron microscopy (TEM) of the isolated fractions confirmed the presence of heterogeneously sized vesicles, in line with the size distributions obtained by NTA. Notably, only the gradient-based method yielded relatively pure vesicle preparations. In contrast, filter-based and SCF methods displayed contamination with cellular debris or protein aggregates, while the bead-based approach was associated with the presence of lipid structures (Figure 2).

**Figure 2:**
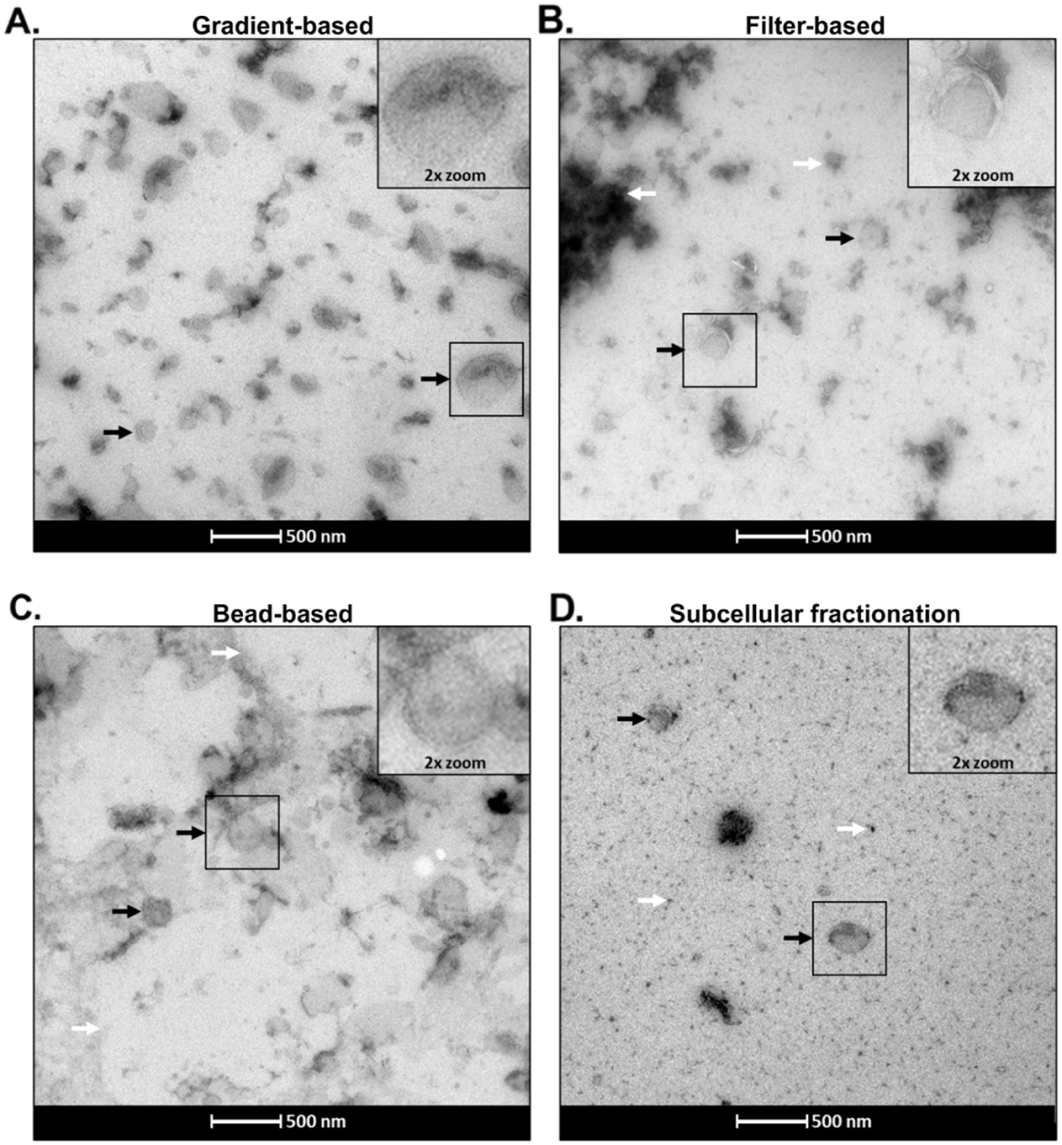
Transmission electron microscopy (TEM) of isolated fractions. Panels A–D show TEM images of the four isolated fractions. Insets display magnified views of the regions outlined in black squares. Black arrows indicate identified representative isolated vesicles, and white arrows denote areas of contamination.

### Lysosome Quantification and Membrane Integrity Assessment

To determine the number of isolated lysosomes and assess their membrane integrity, spectral flow cytometry analysis was performed after combined staining of LysoTracker Deep Red (LTDR) and Calcein AM. LTDR identified acidic lysosomes, while Calcein AM indicated intact organelles (Figure 3A, Figure S1). The median number of lysosomes isolated per cell was highest with the SCF method (27.2), followed by gradient-based (5.4), filter-based (3.8), and bead-based (2.6) methods (*p*-value = 0.02 for SCF vs bead-based; Figure 3B). However, SCF also showed the greatest intra-assay variability. Regarding membrane integrity, the SCF procedure yielded the highest proportion of intact lysosomes (46.5%), followed by the bead-based (42.8%), gradient-based (24.0%), and filter-based (17.6%) methods, with SCF again displaying the largest variability (Figure 3C). The filter-based method resulted in the highest proportion of nonintact lysosomes (68.9% ± 17.5), significantly more than the gradient-based (25.6% ± 32.2, *p*-value = 0.006), bead-based (29.4% ± 45.9, *p*-value = 0.005), and SCF-based (34.1% ± 38.1, *p*-value = 0.005) approaches. Additionally, the filter-based method yielded the lowest percentage of intact nonlysosomes (0.1% ± 0.1), significantly less than the other methods.

**Figure 3:**
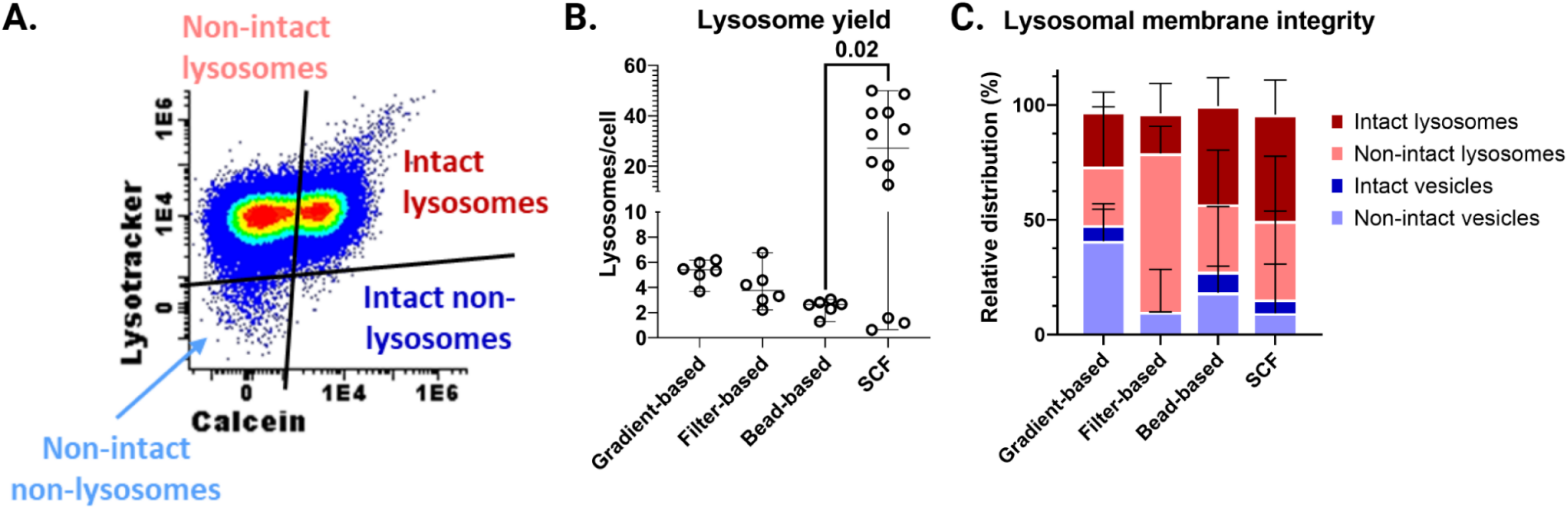
Lysosome quantification and membrane integrity. **A)** Gating strategy used to identify (intact) lysosomes and nonlysosomes, with a representative example from the filter-based method. **B)** Quantification of isolated lysosomes per cell, shown as median with range. Circles represent technical replicates. **C)** Flow cytometry analysis assessing lysosome membrane integrity, presented as the relative distribution (%) with median and range. Each method includes six technical replicates, except for SCF, which includes 12 replicates. SCF, subcellular fractionation. Statistical significance: *p* < 0.05 and FDR 5%. vs ∗ gradient-based, § filter-based, ¶ bead-based, # SCF. For statistical analysis, the nonparametric Kruskal–Wallis test was used and corrected for multiple comparisons using the method of Benjamini and Hochberg.

### Proteomic Characterization of Isolated Lysosomes

Protein profiling of isolated lysosomes was performed by subjecting lysosomes obtained from the four different isolation approaches, alongside total THP-1 cell lysate, to LC–MS/MS proteomics characterization. Each sample yielded the identification of approximately 4,700 proteins, with protein identification criteria requiring at least two unique peptides and a Mascot score of ≥20. All isolation methods produced similar results in terms of the proteins detected and their primary subcellular location, as annotated in the UniProt database (Figure 4A). Although no unique proteins were exclusive to any specific isolation method, differences emerged in the relative abundance of proteins across the samples. Comparative analysis between the isolated fractions and the total cell lysate revealed distinct enrichment patterns, calculated based on the protein abundance in the purified fraction compared to the total cell lysate as a reference value. Both the gradient- and bead-based isolation methods resulted in a marked enrichment of lysosomal proteins, with median normalized abundances of 4,295 and 2,988, respectively, compared to 1,126 in the total lysate (both *p*-values <0.0001). In contrast, the filter-based and SCF methods did not show similar enrichment, with normalized abundances of 1,810 and 1,327, respectively (Figure 4B and 4M).

**Figure 4:**
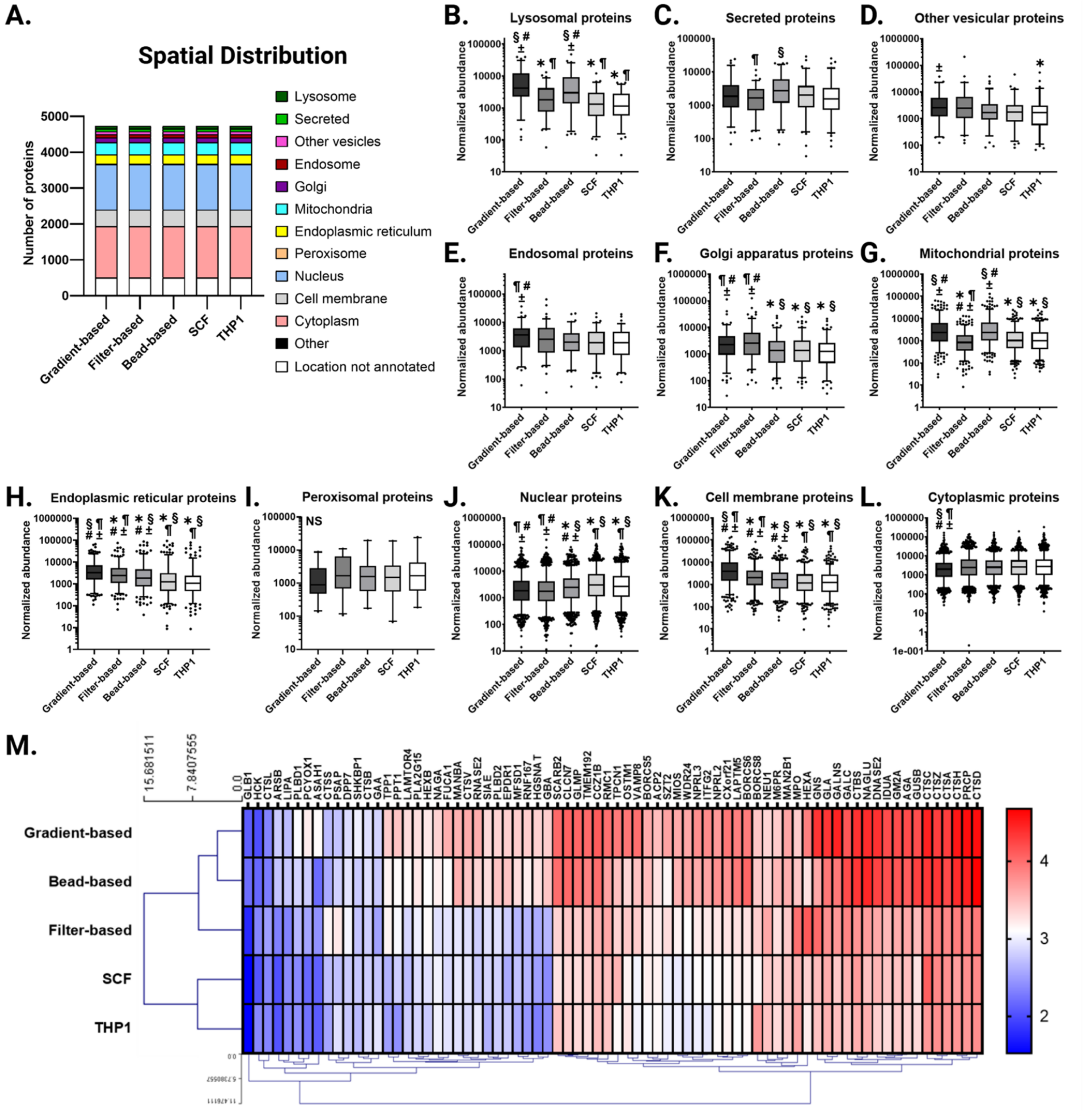
Proteome profile of isolated fractions. A) Overlap of proteins identified across all four isolation methods, categorized by their primary subcellular location (UniProt annotations). B–L) Normalized abundance of proteins grouped by subcellular location (UniProt annotations). Boxes represent the median with interquartile range, and whiskers denote the 5th-95th percentiles. M) Hierarchical clustering (Euclidean distance, average linkage) of log10-transformed normalized abundances for lysosomal proteins. Protein selection criteria: ≥ 2 unique peptides per protein, Mascot score ≥ 20. SCF: subcellular fractionation. Statistical thresholds: *p* < 0.05 and FDR 5%. Symbols denote comparisons: ∗ vs gradient-based, § vs filter-based, ¶ vs bead-based, # vs SCF, ± vs THP1 lysate. For statistical analysis, the nonparametric Kruskal–Wallis test was used and corrected for multiple comparisons using the method of Benjamini and Hochberg.

When examining proteins from other subcellular compartments, the SCF method displayed protein abundances largely comparable to those found in the total THP-1 lysate, except for nuclear proteins, for which a slight increase was noted (median normalized abundance: 3,089 vs 2,688), although this difference was not statistically significant (Figure 4C–L). In contrast, the other isolation methods showed significant variations in protein abundance relative to the total lysate, indicating enrichment of specific cellular compartments. Notably, the gradient-based method showed the highest enrichment of endosomal, endoplasmic reticular, other vesicular, and membrane proteins, with median normalized abundances of 3,540 (vs 1,928, *p*-value = 0.01), 3,259 (vs 1,066, *p* < 0.0001), 2,530 (vs 1,704, *p* = 0.05), and 3,937 (vs 1,193, *p* < 0.0001), respectively. The filter-based approach resulted in the greatest abundance of Golgi apparatus proteins (2,542 vs 1,276, *p* < 0.0001), while the bead-based method was associated with significant enrichment of mitochondrial proteins (2,381 vs 999, *p* < 0.0001).

### Cross-Contamination of Isolated Fractions with Cell Organelles

To evaluate cross-contamination of isolated fractions with other cellular organelles, Western blot (WB) analysis was conducted using six antibodies targeting organelle-specific proteins: COX4 (mitochondria), GM130 (Golgi apparatus), CLNX (endoplasmic reticulum), EEA1 (endosomes), ABCD3 (peroxisomes), and CTSB (lysosomes). A total THP-1 cell lysate was included as a reference for the protein abundance. All isolation procedures showed some degree of cross-contamination with other organelles (Figure 5 and Figure S2), as evidenced by similar abundance patterns in both WB and MS data. Notably, the SCF method exhibited a minimal variation in protein abundance relative to the total cell lysate. The only exceptions were ABCD3, which was enriched in the SCF fraction, and EEA1, which was more abundant in the total THP1 lysate. In contrast, the filter-based approach showed higher levels of all tested proteins, except the mitochondrial marker COX4. Both bead- and gradient-based methods resulted in coisolation of proteins from mitochondria, endoplasmic reticulum, and lysosomes, as indicated by increased COX4, CLNX, and CTSB abundance; however, the bead-based method also showed enrichment of peroxisomes. The bead-based method displayed the highest enrichment of the lysosomal protein CTSB, followed by the gradient- and filter-based methods, consistent with the MS data (Figure 4M and 5F).

**Figure 5:**
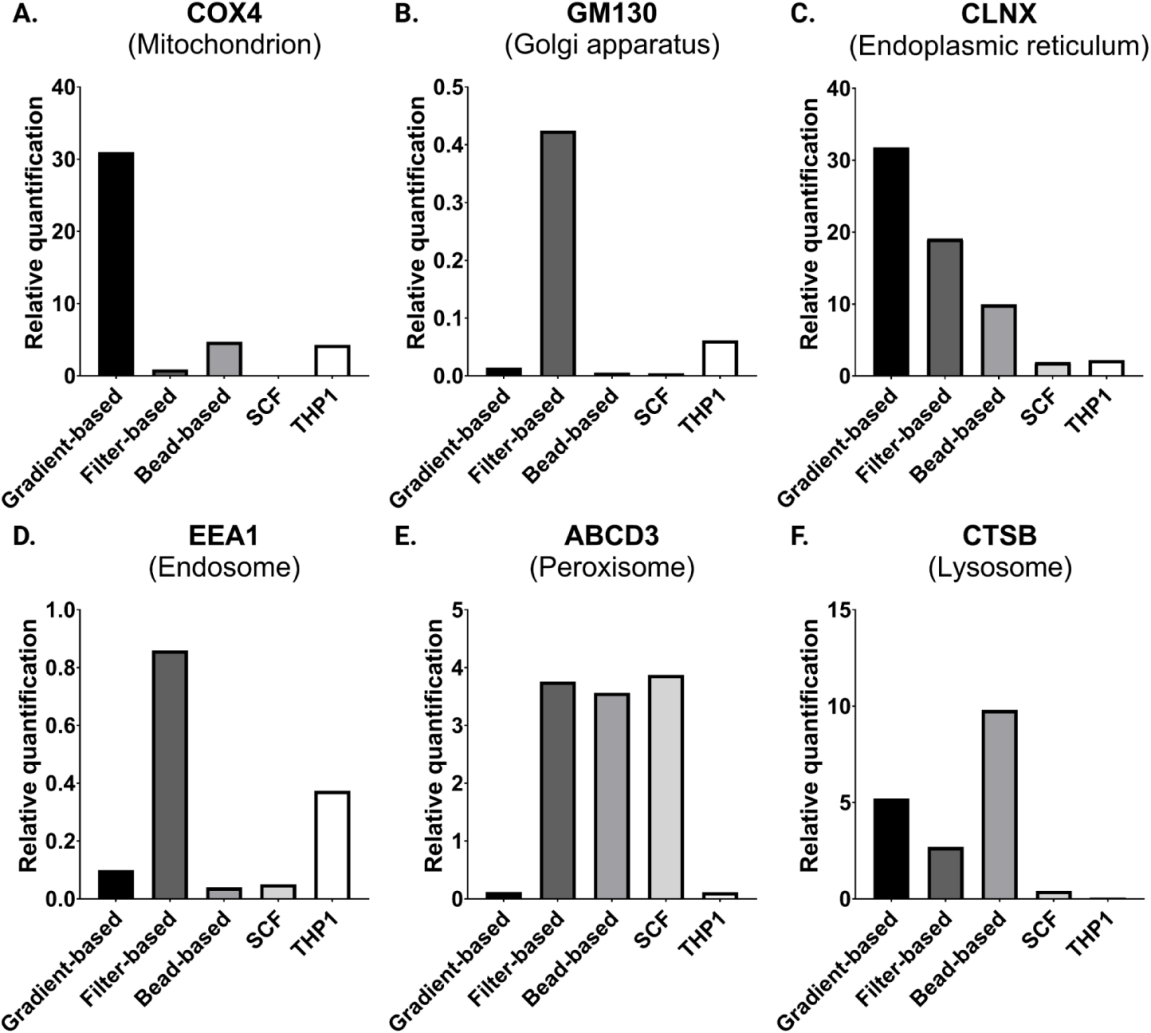
Organelle cross-contamination across lysosome isolation methods. Western blot analysis of selected organelle-specific proteins. Protein levels were quantified relative to the protein ladder. For Western blotting, 10 μg of protein was loaded for **A)** COX4, **B)** GM130, and **C)** CLNX, and 20 μg of protein was loaded for **D)** EEA1, **E)** ABCD3, and **F)** CTSB. SCF, subcellular fractionation.

## Discussion

In recent years, lysosomes have gained increasing recognition as key players in the pathogenesis of numerous diseases, including neurodegenerative disorders, cancer, and cardiovascular conditions.^8–15^ Their dual role as therapeutic targets and sources of clinically relevant biomarkers has underscored the need for robust and reliable lysosome isolation methods, particularly for application in translational and clinical research.^16,19^ However, the low abundance of lysosomes within whole-cell extracts challenges their purification,^22,23^ particularly for downstream applications such as proteomics, biomarker discovery, and functional assays. In this context, our study provides systematic, multimodal benchmarking of four widely used lysosome isolation methods: density gradient-based, filter-based, magnetic bead-based, and SCF, to offer practical guidance tailored to clinical research needs. In our analytical approach, we included the evaluation of six critical parameters: quantitative yield and size distribution profiling, ultrastructural morphological evaluation, assessment of lysosomal membrane integrity, proteomic purity analysis, and screening for cross-contamination with other organelles using NTA, TEM, spectral flow cytometry, LC–MS/MS, and Western blot (WB) methodologies. This multifaceted approach not only quantified lysosome recovery but also assessed their integrity, required for functional competence, and isolation purity, providing a comprehensive framework for identifying method-specific advantages and pitfalls in clinically relevant workflows.

The selection of an appropriate cell line for the assessment of lysosome isolation methods is paramount, particularly when aiming for clinical translation. While cell lines such as HEK293 have been employed in previous studies^48,49^ and offer certain experimental advantages, the use of the THP-1 monocytic cell line provides a more physiologically and pathologically relevant model for numerous clinical applications, especially those involving immune responses or lysosomal storage disorders. THP-1 cells, derived from human monocytic cells, closely mimic the behavior of blood-derived monocytes and macrophages,^50–52^ which are readily accessible in clinical settings for diagnostic or prognostic purposes (e.g., as peripheral blood mononuclear cells, or PBMCs). This ensures that methods performing well with THP-1 cells are more likely to be directly translated to real patient samples. Furthermore, THP-1 cells are extensively utilized to model inflammatory responses *in vitro*, a critical consideration given the increasing implication of lysosomal dysfunction in inflammatory diseases like atherosclerosis, rheumatoid arthritis, and inflammatory bowel disease.^8–15^ The usage of cell types that lack the specialized phagocytic and immune responses, unlike THP-1 cells, might ultimately show differences in lysosomal composition, dynamics, and responses to stimuli that may not accurately mirror those required for understanding human disease. Furthermore, this study was performed using nondifferentiated THP-1 cells, which were intentionally selected as a stringent test system for lysosome isolation. As these cells grow in suspension, they lack substrate-mediated cytoskeletal tension and therefore display distinct cortical mechanics compared to adherent cell lines.^53,54^ These features are known to affect the efficiency and reproducibility of shear-based lysis and homogenization, often requiring cell type specific adjustment.^55,56^ Additionally, unlike differentiated macrophages or secretory models that contain larger and more abundant lysosomes, undifferentiated THP-1 cells contain relatively small (fewer than 10% exceeding 0.5 μm^2^)^57^ and less abundant lysosomes.^58,59^ As a result, lysosome isolation methods are challenged more strongly in this model, and approaches that perform well with THP-1 cells are likely to perform at least as well in cell types with larger or more abundant lysosomes. While absolute yields and optimal parameters may vary among cell types, the key trade-offs observed (i.e., recovery, integrity, cross-contamination, enrichment) arise from physicochemical principles inherent to each separation strategy and therefore are broadly conserved. Consistent with this, a recent comparative study^60^ employing the bead-based isolation method across different cell lines reported limited variability in the yield, organelle cross-contamination, and lysosomal protein identification rates, supporting method transferability. We note, however, that certain biological features have the potential to influence the efficacy of isolation steps and may require cell type-specific optimization. For instance, variations in cytoskeletal organization and membrane shear resistance may affect lysis, lipid droplet content can alter gradient buoyancy, high endoplasmic reticulum content may increase cross-contamination, and phagocytic ability impacts bead uptake. These factors may shift the absolute performance of a given method but are unlikely to change the relative ranking of the methods established here. Overall, by evaluating the performance of the different isolation approaches in a challenging cell model, our findings provide a robust framework for selecting lysosome isolation approaches across diverse cellular systems.

Lysosome recovery yield is particularly crucial in contexts such as high-throughput drug screening, biobanking, and large-scale biomarker discovery,^16,19^ where obtaining sufficient material from limited or precious patient samples is essential. Furthermore, accurate assessment of size distribution is vital for studies focused on lysosomal subpopulations, vesicle trafficking, or the biophysical properties of lysosomes, as even subtle alterations in size can reflect underlying changes in disease state or cellular stress.^61,62^ In our comparative analysis, we observed marked differences in the lysosome yield among the evaluated methods. NTA revealed that both SCF and gradient-based methods produced the highest numbers of particles. However, while SCF was associated with greater variability, the gradient-based approach demonstrated a narrower size distribution and superior reproducibility. In contrast, both filter-based and magnetic bead-based methods yielded lower numbers of particles per cell, with the latter nonetheless offering good reproducibility. Despite this, numerous studies use the magnetic bead-based approach to isolate lysosomes from clinical samples.^30,32,63^ Since the NTA evaluation approach cannot distinguish between lysosomes and other particles present in the sample, LysoTracker staining was used to more specifically evaluate lysosome yield by flow cytometry, depicting the same trend as NTA, but reporting lower numbers of lysosomes per cell (2–40 lysosomes/cell range across methods). Notably, a significant variation was observed across the isolation methods, impacting both the yield (lysosomes per cell) and the integrity (intact vs ruptured vesicles). In this context, it is important to know that in order to truly evaluate the reproducibility of each method, different technical replicates were performed by distinct operators. Specifically, the SCF method exhibited high variability in both the total yield and the integrity profile of the isolated lysosomes. The observed variability likely reflects the operator-dependent nature of the manual fractionation steps. Because technical replicates were performed by different operators, subjective differences in fluid handling significantly impacted outcomes. Specifically, the manual aspiration of supernatant from a loose pellet in viscous 20% glycerol buffer may have introduced interoperator inconsistencies in recovery volume. Furthermore, variations in pipetting vigor during the resuspension of the lysosome-enriched pellet resulted in distinct shear stress profiles, leading to fluctuations in membrane integrity and yield across replicates. In this context, these sources of variability could be substantially reduced through protocol standardization (e.g., fixed stroke counts for pipetting) or partial automation (e.g., using controlled aspiration devices, low-shear resuspension tools, or semiautomated fraction collectors), which would reduce operator-dependent differences and improve reproducibility for laboratories implementing the SCF approach. In contrast, the magnetic bead-based method demonstrated stable and reproducible yields (number of lysosomes per cell) but still showed notable variability in the distribution of intact versus ruptured vesicles. This suggests that while the magnetic process ensures consistent isolation efficiency, the observed variation is likely due to the increased fragility of a subset of lysosomes coupled with the cumulative mechanical stress or varying processing times associated with a longer, multistep protocol.

The preservation of ultrastructural morphology and membrane integrity in isolated lysosomes is essential for ensuring the validity of downstream functional and mechanistic studies, such as those investigating lysosomal enzyme activity, ion homeostasis, or vesicle fusion events, as compromised membranes can lead to leakage of luminal contents and artifactual results.^64,65^ Moreover, research focused on lysosome-mediated signaling pathways, drug delivery, or the development of enzyme replacement therapies requires preparations that closely mimic the native state of lysosomes within cells.^49,66,67^ In this regard, flow cytometry analysis provided critical insights into the membrane integrity of the lysosomes isolated by each method. Using dual staining with LysoTracker and Calcein, the magnetic bead-based and SCF methods consistently exhibited the highest proportion of membrane-intact, acidic vesicles, as previously reported by Singh et al.,^35^ indicating that these protocols most effectively preserve the physiological state of lysosomes during isolation. In contrast, gradient-based and, particularly, filter-based approaches yielded a significantly larger fraction of membrane-compromised lysosomes. Our results were consistent with those of Singh et al.;^35^ however, they reported much more lysosome damage with these types of protocols, which might be due to differences in the protocol itself and/or the method of calculating such recovery. Regarding the latter, most studies determine the percentage of intact lysosomes by estimating the different activity levels of a lysosomal protein (e.g., β-hexosaminidase) with and without disrupting the membrane using a detergent (e.g., Triton X-100) as a positive control.^68^ Here, we employed flow cytometry analysis using LysoTracker and Calcein markers, which provide a more reliable assessment of lysosomal membrane integrity without relying on one specific lysosomal protein and thus less susceptible to potential detection issues due to mutation.

Another central challenge in lysosome isolation is minimizing cross-contamination with other organelles, as the presence of nonlysosomal vesicles can compromise data interpretation. This is particularly critical for studies aiming to characterize the lysosomal proteome, identify disease-specific biomarkers, or investigate lysosome-specific signaling pathways, where even low-level contamination can lead to false positives or obscure subtle biological differences.^3,16,18,19,69^ Furthermore, functional studies, such as those assessing lysosomal enzyme activity, drug response, or vesicle trafficking, require highly pure lysosome preparations to ensure that the observed effects are truly lysosome specific. Thus, our study combined ultrastructural assessment by TEM with quantitative proteomics and WB to comprehensively evaluate the purity of the lysosome preparations obtained. Proteomics data revealed that bead-based and SCF methods consistently achieved the highest lysosomal purity, with minimal detection of mitochondrial, Golgi apparatus, endoplasmic reticulum, or endosomal markers. In contrast, lysosome fractions isolated by filter-based approaches exhibited significant contamination, as evidenced by the enrichment of nonlysosomal proteins in proteomic and WB analyses. Only mitochondrial proteins appeared to not be coisolated with this method. Of note, all methods besides the gradient-based approach showed cross-contamination with peroxisomal proteins. Nevertheless, gradient-based methods also displayed moderate coisolation of nonlysosomes, which aligns with previous studies.^24,25^

The performance of each isolation method (yield, purity, and integrity) is directly determined by its underlying physicochemical and biochemical separation mechanisms. Gradient-based isolation relies on the native buoyant density. While foundational, purity is inherently limited by coisolation with density-matched organelles (e.g., endosomes, peroxisomes). The high centrifugal forces required also lead to compromised membrane integrity, limiting its use in functional assays. Filter-based isolation utilizes size exclusion. Its simplicity and speed are offset by poor purity, as vesicle size heterogeneity allows numerous nonlysosomal vesicles to coisolate. Furthermore, the passage through narrow pores generates shearing forces, causing extensive membrane damage and low integrity. Bead-based isolation employs a biochemical principle by utilizing phagocytosis to tag lysosomes with magnetic beads (forming phagolysosomes). While yielding high purity, this mechanism fundamentally alters the vesicle’s native state, imposing significant limitations on assessing true lysosomal function or composition. SCF, rooted in differential centrifugation, separates based on the sedimentation coefficient. Its high yield and cost-effectiveness are achieved through minimal, rapid steps. However, its purity and reproducibility are highly variable, mainly operator-related.

Overall, when selecting a lysosomal enrichment strategy, it is essential to align the method with the specific experimental objectives, as each approach offers distinct advantages and limitations in terms of yield, purity, intactness, and reproducibility. Table 1 shows a summary of the obtained results and could guide researchers in selecting the optimal isolation method. Gradient-based methods are notable for their ability to produce large amounts of particles with high reproducibility. However, these techniques yield a relatively limited number of intact lysosomes and exhibit moderate cross-contamination, making them less suitable for functional studies requiring absolute membrane integrity. This limitation may help explain the findings of Xu et al.,^24^ who were unable to find the GFAP protein within the lysosomal compartment—potentially due to the use of disrupted lysosomes in their analyses. Likewise, other studies could benefit from alternative isolation strategies to enhance lysosomal proteome coverage.^25^ Remarkably, the analysis by Bové et al.,^70^ focused on the permeabilization of lysosomal membranes as a major pathogenic event in Parkinson’s disease, might have achieved more robust results by selecting a method that better preserves lysosomal integrity (e.g., magnetic bead-based isolation), thereby ensuring faithful representation of lysosomal membrane physiology in their experimental model. Also, gradient-based protocols require specialized equipment such as ultracentrifuges and dounce homogenizers, which may not be readily available in clinical laboratories, and the procedures themselves are labor-intensive. Despite this, numerous studies rely on density-based centrifugations to enrich lysosomes.^26–28,71,72^ Magnetic bead-based isolation, while maintaining good membrane integrity in approximately half of the recovered lysosomes and keeping cross-contamination at low levels, suffers from a lower particle yield. This limitation can restrict its application in resource-intensive settings, such as biobanking or large-scale analyses. Nevertheless, because this method preserves lysosome integrity, studies on lysosomal contents can be achieved, as demonstrated by Nackenoff et al.^31^ who showed that lysosomes (isolated with dextran-coated iron nanoparticles) in dystrophic neurites of Alzheimeŕs disease patients appear to function suboptimally compared to lysosomes from neuronal cell bodies. Likewise, Tharkeshwar and collaborators^32^ performed lipidomics and proteomics studies on SPION-isolated lysosomes from HeLa cells to study the Niemann-Pick disease type C but required large cell amounts. This disease was also studied at the lysosomal level by Kosicek et al.,^61^ who revealed the N-glycome profiling of the lysosomal glycocalyx and reported specific changes in the lysosomal glycocalyx in *NPC1*-null cells, suggesting that these changes might contribute to lysosomal dysfunction. Despite all of the positive features of this method, it relies on phagocytosis, restricting its use to phagolysosome isolation, which may also alter the physiological state and proteomic profile of the lysosomes. In line with this, Uzhytchak et al.^73^ reported potential alteration of subcellular signaling and induction of oxidative stress after accumulation of iron oxide-based nanoparticles, which are used for most magnetic bead-based isolation methods. Additionally, nanoparticle uptake can modulate endolysosomal physiology (e.g., pH, enzyme activity, or membrane permeability) depending on particle composition, coating, dose, and exposure time. Dextran-coated superparamagnetic iron-oxide particles are widely used and are generally well tolerated, but they are metabolized within lysosomes and can alter lysosomal chemistry at high doses or with short chase periods.^74,75^ Furthermore, reduced cathepsin activity and altered lysosomal function for some iron-oxide formulations in sensitive cell types have also been reported.^76^ These effects could be potentially minimized by using the lowest bead dose that yields acceptable recovery, increasing the chase period to allow phagolysosome maturation, or validating lysosomal pH/protease activity in bead-loaded vs control cells. Therefore, studies employing this method should be carefully reevaluated, as their results may not accurately reflect the physiological state of lysosomes. The approach is also costly and time-consuming and requires specialized magnetic separation equipment, making it the least desirable option for routine use. On the other hand, SCF offers the highest lysosome yield and effectively preserves membrane integrity, with relatively low cross-contamination (only observed for peroxisomal proteins). However, significant variability between preparations limits its reliability for studies requiring high reproducibility. On the positive side, SCF is the most cost-effective and least time-consuming method, with scalability that accommodates varying amounts of starting material.^77^ Finally, filter-based methods, while rapid and straightforward, consistently underperform all of the evaluated parameters. They yield low particle numbers, show extensive membrane damage, and are associated with high levels of contamination from nonlysosomal vesicles (except mitochondria), rendering them unsuitable for studies demanding functional or compositional fidelity. These poor features correlate with the limited number of peer-reviewed studies focusing on filter-based lysosome isolation approaches.

**Table 1:** Summary of Key Features of the Lysosome Isolation Methods Tested in This Study^a^

	Method	Gradient-based	Filter-based	**Magnetic bead-based**	**Subcellular fractionation**
**METHOD FEATURES**	*Description*	Density-based isolation using an ultracentrifuge	Size-based using a spin-column	Magnet-based isolation after magnetic bead phagocytosis	Differential centrifugation using varying lysis buffers
	*Available commercial kit*	Lysosome Enrichment Kit for Tissues and Cultured Cells (Thermo Fisher, cat 89839)	Minute Lysosome Isolation Kit for Mammalian Cells/Tissues (Invent Biotech, cat LY-034)	DexoMAG 40 (Liquids Research, cat DEXOMAG-C)	-
	*Minimum sample size (according to manufacturer)*	50–200 mg of cultured cells or tissue	2.5–3 x10^7^ cultured cells or 20–30 mg of tissue	2x T75 or 1x T175 culture flask	Not specified
	*Special equipment needed*	•Sonicator or Dounce homogenizer	-	•Miltenyi QuadroMACS magnetic separator	Tube rotator
		•Ultracentrifuge		•Dounce homogenizer	
**YIELD**	*No. of isolated vesicles/cell – according to NTA (median±range)*	**HIGH**	**LOW**	**LOWEST**	**HIGHEST**
		1,630.2±1632.3	228.2±265.6	27.8±35.2	2.356.3±7,461.7
	*Size range (in nm; median D50 [median D10-median D90])*	**LOWEST**	**HIGHEST**	**HIGH**	**LOW**
		159.5 [108.2–250.8]	169.9 [105.6–308.5]	168.9 [94.1–273]	150.0 [87.1–244.9]
	*No. of isolated lysosomes/cell – according to FCM (median±range)]*	**MODERATE**	**MODERATE**	**LOWEST**	**HIGHEST**
		5.4±2.5	3.8±4.5	2.6±1.8	27.2±49.4
**MEMBRANE INTEGRITY**	*No. of isolated intact lysosomes/cell—according to FCM [% of total vesicles]*	**LOW**	**LOWEST**	**HIGH**	**HIGHEST**
		2.0 [24.1]	0.8 [17.6]	1.8 [42.8]	12.8 [46.5]
**REPRODUCIBILITY**	*FCM intra-assay reproducibility [% CV]*	**HIGHEST**	**LOW**	**LOW**	**LOWEST**
		16.81	39.52	25.35	72.28
**CONTAMINATION**	*Fraction contamination according to TEM images*	None	Protein aggregates	Lipid structures	Protein aggregates
	*Organelle cross-contamination according to MS and WB data*	Endosomes, ER, membrane, other vesicles	Golgi, ER, endosomes, peroxisomes	Mitochondria, ER, peroxisome	Nuclear, peroxisome
**COSTS**	*Processing cost per sample*	∼€35	∼€30	∼€120	∼€3
**TIME**	*Processing time*	∼5 h	∼2.5 h	∼43 h	∼1.5 h

a FCM, flow cytometry; CV, coefficient of variation; TEM, transmission electron microscopy; MS, mass spectrometry; WB, Western blot; ER, endoplasmic reticulum; h, hour.

Our multiapproach assessment also highlights the importance of rigorous method validation. While MS provides comprehensive data on lysosome enrichment and integrity, it is labor-intensive and not practical for routine validation. Western blotting and TEM offer valuable complementary information but also have their own limitations, such as semiquantitative output and sample preparation complexity. Rapid techniques such as NTA can estimate vesicle yield but lack specificity for lysosomes unless combined with additional markers, whereas flow cytometry enables high-throughput assessment of lysosome purity and integrity when using appropriate staining protocols. Thus, integrating multiple complementary validation techniques is essential to ensure accurate characterization of lysosomal procedures, as shown herein. In this context, flow cytometry stands out as a strong candidate for overall quality control in lysosome isolation. It offers quick, quantitative insights into the yield, membrane integrity, and major cross-contamination. While it does not provide absolute purity of the proteome/lipidome or detailed ultrastructural morphology of lysosomes, its broad availability in clinical laboratories and the high reproducibility demonstrated in our study, even when experiments were performed by different operators, highlight its potential for standardized implementation across different research and clinical settings.

Another practical consideration is the amount of starting material required. In this study, we standardized the input to 4 × 10^7^ cells to allow for direct comparison across methods. However, such large sample sizes may not always be feasible, especially in clinical settings. For instance, tissue biopsies are typically restricted in size and often require tissue-specific adaptations, as reported for the gradient-based isolation of lysosomes from skeletal muscle.^78^ Filter- and gradient-based protocols generally require large cell numbers, while SCF and bead-based approaches may be more adaptable to smaller sample sizes, although further optimization would be required. Finally, it is noteworthy that newer approaches have been developed based on the immunoprecipitation of lysosomes using the endogenous integral lysosomal membrane protein TMEM192, available in both tagged^48,49,79,80^ and untagged^36^ formats. However, the tagged version requires genetic modification to introduce the tag, which is not feasible for patient samples. While the untagged approach overcomes this limitation, it still faces challenges related to lysosome yield and purity. Therefore, further optimization and development of these methods are needed to enhance their applicability, especially in clinical settings. In addition, the known variability of TMEM192 expression across different human tissues poses a significant difficulty.^81,82^ This variability necessitates extensive pretesting of protein levels, making TMEM192-based methods less suitable for diverse clinical samples. Nevertheless, published data suggest that immunoprecipitation-based techniques offer high purity and should be considered for contexts where genetic editing is feasible,^83^ such as established laboratory cell lines, or samples with high and stable TMEM192 expression. Furthermore, studies employing a combined strategy based on the sequential enrichment of lysosomes with SPIONs and TMEM-IP have reported increased purity of lysosomal fraction.^84^

Multimodal benchmarking of different approaches for lysosome isolation highlighted a trade-off among key performance metrics: yield is maximized by SCF, purity and reproducibility are best achieved through gradient-based methods, and higher specificity is obtained by employing magnetic bead isolation. While the fastest and most cost-effective techniques (i.e., SCF and filter-based isolation) offer clear economic and temporal advantages, they require a significant sacrifice in reproducibility and overall sample quality. This deficit is most pronounced in membrane integrity, where the physical shear stress inherent to filter-based mechanisms proves detrimental compared to the superior preservation afforded by the milder buffer-based lysis used in subcellular fractionation. Given the physicochemical trade-offs observed, no single method serves as a universal solution. Instead, the choice of isolation is heavily dependent on the specific downstream application. Accordingly, for quantitative proteomics and/or lipidomics studies, where data reliability is paramount, gradient-based isolation is the best approach. Its reliance on isopycnic centrifugation to separate organelles based on density equilibrium yields the highest intra-assay reproducibility, ensuring a proteomic signature free of aggregate contamination. By utilizing mild lysis buffers and the absence of high-shear forces, SCF preserved membrane integrity and ensured high yield, emerging as the most cost-effective and time-efficient method. Consequently, it is the optimal choice for large-scale screening or biobanking, or even functional enzymatic assays, where purity is secondary to biomass recovery (e.g., initial biomarker discovery from large sample sets), provided that the inherent variability is effectively managed or deemed acceptable. Conversely, the filter-based method, with its quick processing and moderate lysosome yield, may be appropriate for focused enzymatic or functional assays where specific organelle populations are targeted postisolation, provided its cross-contamination is accounted for. However, the shear forces generated by passing cellular lysates through porous matrices result in low membrane integrity and significant protein aggregate contamination, rendering it unsuitable for structural or functional assessments requiring organelle integrity. Finally, the bead-based approach, although limited by the low overall yield and lengthy protocol, achieves high specificity and membrane integrity due to its reliance on phagocytosis, allowing the targeted isolation of phagolysosomes. This renders it ideal for highly targeted downstream analyses, such as validating surface markers via WB or studying unique vesicle subpopulations, where excluding mitochondrial and peroxisomal contaminants is paramount, or for research targeting specific uptake mechanisms. However, it is important to realize that potential alteration of lysosome physiology may occur due to the phagocytosis process, and the isolation is restricted to phagolysosomes. A summarizing decision tree can be found in Figure S3.

In summary, a thorough evaluation of lysosome isolation methods is absolutely critical for both fundamental research and clinical applications. These organelles play vital roles in cellular processes and disease, and any compromise during isolation can lead to misleading results. Key parameters for assessment include yield, which ensures sufficient material for discovery in research and robust analysis of scarce clinical samples. Integrity and purity (cross-contamination) are crucial for accurately attributing molecular components to lysosomes, preserving their contents for functional studies, and identifying unbiased biomarkers, all of which prevent false results and ensure the reliability of the findings. Furthermore, intra-assay reproducibility is fundamental for consistent data in research and indispensable for reliable diagnostic and monitoring tools in clinical settings. Finally, considering the heterogeneity of the vesicle size ensures the capture of diverse lysosomal subpopulations, which is essential for understanding complex biology and detecting disease-specific morphological changes. Ignoring any of these aspects can lead to incorrect conclusions in basic science and, more significantly, unreliable diagnostics and ineffective therapies in clinical practice. Recognizing that a complete evaluation of all these parameters is not always feasible, particularly in a clinical laboratory setting, the comprehensive benchmarking presented in this study offers a valuable reference, as it will help guide researchers and clinicians in selecting the most appropriate lysosome isolation strategies, tailored to their specific goals.

## Conclusion

This study provides a systematic benchmarking of four lysosome isolation strategies, revealing inherent trade-offs among yield, integrity, purity, and reproducibility driven by their underlying separation principles. No single method is universally optimal; instead, the method selection must be guided by the intended downstream application. By integrating complementary analytical approaches in a clinically relevant and challenging monocytic model, our work offers a practical framework to support the informed selection of lysosome enrichment strategies, facilitating reliable lysosomal analyses in both research and translational settings.

## Supplementary Material



## Notes

The workflow for flow cytometry data analysis (Figure S1), the original Western blot bands (Figure S2), and a summarizing decision tree to aid researchers in selecting the most optimal lysosome enrichment method (Figure S3) (PDF)

## References

[1] De
Duve C., Pressman B. C., Gianetto R., Wattiaux R., Appelmans F. (1955). Tissue fractionation
studies. 6. Intracellular distribution patterns of enzymes in rat-liver
tissue”. Biochem. J..

[2] Saftig P., Klumperman J. (2009). Lysosome biogenesis
and lysosomal membrane proteins:
trafficking meets function. Nat. Rev. Mol. Cell
Biol..

[3] Schröder B. A., Wrocklage C., Hasilik A., Saftig P. (2010). The proteome of lysosomes. Proteomics.

[4] Ballabio A., Bonifacino J. S. (2020). Lysosomes
as dynamic regulators of cell and organismal
homeostasis. Nat. Rev. Mol. Cell Biol..

[5] Settembre C., Fraldi A., Medina D. L., Ballabio A. (2013). Signals from the lysosome:
a control centre for cellular clearance and energy metabolism. Nat. Rev. Mol. Cell Biol..

[6] Alomari M. (2004). Color Atlas
of Cytology, Histology and Microscopic Anatomy. Ann. Saudi Med..

[7] Yang C., Wang X. (2021). Lysosome biogenesis: Regulation and
functions. J. Cell Biol..

[8] Gros F., Muller S. (2023). The role of lysosomes in metabolic
and autoimmune diseases. Nat. Rev. Nephrol..

[9] Sharma J., di Ronza A., Lotfi P., Sardiello M. (2018). Lysosomes
and Brain Health. Annu. Rev. Neurosci..

[10] Fraldi A., Klein A. D., Medina D. L., Settembre C. (2016). Brain Disorders
Due to Lysosomal Dysfunction. Annu. Rev. Neurosci..

[11] Steffan J. J., Williams B. C., Welbourne T., Cardelli J. A. (2010). HGF-induced invasion
by prostate tumor cells requires anterograde lysosome trafficking
and activity of Na+-H+ exchangers. J. Cell Sci..

[12] Yang S., Wang X., Contino G., Liesa M., Sahin E., Ying H., Bause A., Li Y., Stommel J. M., Dell’antonio G., Mautner J., Tonon G., Haigis M., Shirihai O. S., Doglioni C., Bardeesy N., Kimmelman A. C. (2011). Pancreatic
cancers require autophagy for tumor growth. Genes Dev..

[13] Davidson S. M., Vander Heiden M. G. (2017). Critical Functions of the Lysosome in Cancer Biology. Annu. Rev. Pharmacol. Toxicol..

[14] Appelqvist H., Wäster P., Kågedal K., Öllinger K. (2013). The lysosome:
from waste bag to potential therapeutic target. J. Mol. Cell. Biol..

[15] Yuan X. M., Li W., Brunk U. T., Dalen H., Chang Y. H., Sevanian A. (2000). Lysosomal
destabilization during macrophage damage induced by cholesterol oxidation
products. Free Radic Biol. Med..

[16] Zhang X., Wu H., Tang B., Guo J. (2024). Clinical, mechanistic, biomarker,
and therapeutic advances in GBA1-associated Parkinson’s disease. Transl. Neurodegener..

[17] Huh Y. E., Usnich T., Scherzer C. R., Klein C., Chung S. J. (2023). GBA1 Variants
and Parkinson’s Disease: Paving the Way for Targeted Therapy. J. Mov. Disord..

[18] Sun C., Zhang N., Hu Q., Xia G. (2023). Ferroptosis-Related
Prognostic Gene LAMP2 Is a Potential Biomarker Differential Expressed
in Castration Resistant Prostate Cancer. Dis.
Markers.

[19] Liu S. P., Li X. M., Liu D. M., Xie S. H., Zhang S. B., Li Y., Xie Z. F. (2022). LAMP2 as a Biomarker
Related to Prognosis and Immune
Infiltration in Esophageal Cancer and Other Cancers: A Comprehensive
Pan-Cancer Analysis. Front. Oncol..

[20] Luzio J. P., Hackmann Y., Dieckmann N. M., Griffiths G. M. (2014). The biogenesis
of lysosomes and lysosome-related organelles. Cold Spring Harb. Perspect. Biol..

[21] Klumperman J., Raposo G. (2014). The complex ultrastructure
of the endolysosomal system. Cold Spring Harb.
Perspect. Biol..

[22] Au C. E., Bell A. W., Gilchrist A., Hiding J., Nilsson T., Bergeron J. J. (2007). Organellar proteomics
to create the cell map. Curr. Opin. Cell Biol..

[23] Mosen P., Sanner A., Singh J., Winter D. (2021). Targeted Quantification
of the Lysosomal Proteome in Complex Samples. Proteomes.

[24] Xu S., Sleat D. E., Jadot M., Lobel P. (2010). Glial fibrillary acidic
protein is elevated in the lysosomal storage disease classical late-infantile
neuronal ceroid lipofuscinosis, but is not a component of the storage
material. Biochem. J..

[25] Markmann S., Krambeck S., Hughes C. J., Mirzaian M., Aerts J. M., Saftig P., Schweizer M., Vissers J. P., Braulke T., Damme M. (2017). Quantitative Proteome
Analysis of Mouse Liver Lysosomes Provides
Evidence for Mannose 6-phosphate-independent Targeting Mechanisms
of Acid Hydrolases in Mucolipidosis II. Mol.
Cell. Proteomics.

[26] Jinn S., Blauwendraat C., Toolan D., Gretzula C. A., Drolet R. E., Smith S., Nalls M. A., Marcus J., Singleton A. B., Stone D. J. (2019). Functionalization of the TMEM175 p.M393T variant as
a risk factor for Parkinson disease”. Hum. Mol. Genet..

[27] Navarro-Romero A., Fernandez-Gonzalez I., Riera J., Montpeyo M., Albert-Bayo M., Lopez-Royo T., Castillo-Sanchez P., Carnicer-Caceres C., Arranz-Amo J. A., Castillo-Ribelles L. (2022). Lysosomal lipid alterations
caused by glucocerebrosidase deficiency promote lysosomal dysfunction,
chaperone-mediated-autophagy deficiency, and alpha-synuclein pathology. NPJ. Parkinson’s Disease.

[28] Beaumelle B. D., Gibson A., Hopkins C. R. (1990). Isolation and preliminary
characterization
of the major membrane boundaries of the endocytic pathway in lymphocytes. J. Cell Biol..

[29] Biotechnologies, I. . Minute Lysosome Isolation Kit, https://inventbiotech.com/products/minute%E2%84%A2-lysosome-isolation-kit-for-mammalian-cells-tissues, Accessed 13 January 2025.

[30] Diettrich O., Mills K., Johnson A. W., Hasilik A., Winchester B. G. (1998). Application
of magnetic chromatography to the isolation of lysosomes from fibroblasts
of patients with lysosomal storage disorders. FEBS Lett..

[31] Nackenoff A. G., Hohman T. J., Neuner S. M., Akers C. S., Weitzel N. C. S., Ferguson S. M., Mobley B., Bennett D. A., Schneider J. A., Jefferson A. L. (2021). PLD3 is a neuronal lysosomal phospholipase
D associated with β-amyloid plaques and cognitive function in
Alzheimer’s disease. PLoS Genetics.

[32] Tharkeshwar A. K., Trekker J., Vermeire W., Pauwels J., Sannerud R., Priestman D. A., Te Vruchte D., Vints K., Baatsen P., Decuypere J. P., Lu H., Martin S., Vangheluwe P., Swinnen J. V., Lagae L., Impens F., Platt F. M., Gevaert K., Annaert W. (2017). A novel approach
to analyze lysosomal
dysfunctions through subcellular proteomics and lipidomics: the case
of NPC1 deficiency. Sci. Rep..

[33] Le T. S., Takahashi M., Isozumi N., Miyazato A., Hiratsuka Y., Matsumura K., Taguchi T., Maenosono S. (2022). Quick and
Mild Isolation of Intact Lysosomes Using Magnetic-Plasmonic Hybrid
Nanoparticles. ACS Nano.

[34] Díez P., Droste C., Dégano R. M., González-Muñoz M., Ibarrola N., Pérez-Andrés M., Garin-Muga A., Segura V., Marko-Varga G., La Baer J., Orfao A., Corrales F. J., De Las
Rivas J., Fuentes M. (2015). Integration of Proteomics
and Transcriptomics Data Sets for the Analysis of a Lymphoma B-Cell
Line in the Context of the Chromosome-Centric Human Proteome Project. J. Proteome Res..

[35] Singh J., Kaade E., Muntel J., Bruderer R., Reiter L., Thelen M., Winter D. (2020). Systematic Comparison
of Strategies
for the Enrichment of Lysosomes by Data Independent Acquisition. J. Proteome Res..

[36] Saarela D. (2025). Tagless LysoIP for immunoaffinity enrichment of native
lysosomes
from clinical samples. J. Clin. Invest..

[37] Pertoft H., Rubin K., Kjellén L., Laurent T. C., Klingeborn B. (1977). The viability
of cells grown or centrifuged in a new density gradient medium, Percoll­(TM)”. Exp. Cell Res..

[38] de Araùjo, M. E. ; Huber, L. A. ; Stasyk, T. Isolation of endocitic organelles by density gradient centrifugation 2D PAGE: Sample Preparation and Fractionation Springer 2008 317–331 10.1007/978-1-60327-064-9_25 18369872

[39] Arai K., Kanaseki T., Ohkuma S. (1991). Isolation
of highly purified lysosomes
from rat liver: identification of electron carrier components on lysosomal
membranes. J. Biochem..

[40] Islinger M., Wildgruber R., Völkl A. (2018). Preparative free-flow electrophoresis,
a versatile technology complementing gradient centrifugation in the
isolation of highly purified cell organelles. Electrophoresis.

[41] Mathew W. W., Emyr L.-E. (2015). A rapid method for
the preparation of ultrapure, functional
lysosomes using functionalized superparamagnetic iron oxide nanoparticles. Methods Cell Biol..

[42] Varalda M., Antona A., Bettio V., Roy K., Vachamaram A., Yellenki V., Massarotti A., Baldanzi G., Capello D. (2020). Psychotropic
Drugs Show Anticancer Activity by Disrupting Mitochondrial and Lysosomal
Function. Front. Oncol..

[43] Ota K., Okuma T., Lorenzo A., Yokota A., Hino H., Kazama H., Moriya S., Takano N., Hiramoto M., Miyazawa K. (2019). Fingolimod sensitizes EGFR wild-type non-small cell
lung cancer cells to lapatinib or sorafenib and induces cell cycle
arrest. Oncol. Rep..

[44] Ramsby M. L., Makowski G. S. (1998). Differential detergent
fractionation of eukaryotic
cells. Analysis by two-dimensional gel electrophoresis”. Methods Mol. Biol..

[45] van
der Pan K. (2022). Quantitative proteomics of small numbers of closely-related
cells: Selection of the optimal method for a clinical setting. Front. Med..

[46] Hughes C. S., Foehr S., Garfield D. A., Furlong E. E., Steinmetz L. M., Krijgsveld J. (2014). Ultrasensitive proteome analysis using paramagnetic
bead technology. Mol. Syst. Biol..

[47] Hughes C. S., Moggridge S., Müller T., Sorensen P. H., Morin G. B., Krijgsveld J. (2019). Single-pot,
solid-phase-enhanced sample preparation
for proteomics experiments. Nat. Protocols.

[48] Davis O. B., Shin H. R., Lim C. Y., Wu E. Y., Kukurugya M., Maher C. F., Perera R. M., Ordonez M. P., Zoncu R. (2021). NPC1-mTORC1
Signaling Couples Cholesterol Sensing to Organelle Homeostasis and
Is a Targetable Pathway in Niemann-Pick Type C. Develop. Cell..

[49] Laqtom N. N., Dong W., Medoh U. N., Cangelosi A. L., Dharamdasani V., Chan S. H., Kunchok T., Lewis C. A., Heinze I., Tang R. (2022). CLN3 is
required for
the clearance of glycerophosphodiesters from lysosomes. Nature.

[50] Tsuchiya S., Yamabe M., Yamaguchi Y., Kobayashi Y., Konno T., Tada K. (1980). Establishment and characterization
of a human acute monocytic leukemia cell line (THP-1)”. Int. J. Cancer.

[51] Chanput W., Mes J. J., Wichers H. J. (2014). THP-1 cell
line: An in vitro cell
model for immune modulation approach. Int. Immunopharmacol..

[52] Sharma P., Venkatachalam K., Binesh A. (2024). Decades Long Involvement of THP-1
Cells as a Model for Macrophage Research: A Comprehensive Review. Anti-Inflammatory Anti-Allergy Agents Med. Chem..

[53] Pegoraro, A. F. ; Janmey, P. ; Weitz, D. A. Mechanical Properties of the Cytoskeleton and Cells”, Cold Spring Harbor perspectives in biology. 2017, 9(11). a022038 10.1101/cshperspect.a022038. PMC5666633 29092896

[54] Rosenbluth M. J., Lam W. A., Fletcher D. A. (2006). Force microscopy of nonadherent cells:
a comparison of leukemia cell deformability. Biophys. J.

[55] de
Araújo M. E., Lamberti G., Huber L. A. (2015). Homogenization of
Mammalian Cells. Cold Spring Harb. Protoc..

[56] De
Caprio J., Kohl T. O. (2019). Using Dounce Homogenization to Lyse
Cells for Immunoprecipitation. Cold Spring Harb.
Protoc..

[57] Bussi C., Gutierrez M. G. (2024). One size does not fit all: Lysosomes
exist in biochemically
and functionally distinct states. PLoS Biol..

[58] Phuangbubpha P., Thara S., Sriboonaied P., Saetan P., Tumnoi W., Charoenpanich A. (2023). Optimizing
THP-1 Macrophage Culture for an Immune-Responsive
Human Intestinal Model. Cells.

[59] Spano A., Barni S., Bertone V., Sciola L. (2013). Changes on lysosomal
compartment during PMA-induced differentiation of THP-1 monocytic
cells: Influence of type I and type IV collagens. Adv. Biosci. Biotechnol..

[60] Akter F., Bonini S., Ponnaiyan S., Kögler-Mohrbacher B., Bleibaum F., Damme M., Renard B. Y., Winter D. (2023). Multi-Cell
Line Analysis of Lysosomal Proteomes Reveals Unique Features and Novel
Lysosomal Proteins. Mol. Cell. Proteom..

[61] Kosicek M., Gudelj I., Horvatic A., Jovic T., Vuckovic F., Lauc G., Hecimovic S. (2018). N-glycome
of the Lysosomal Glycocalyx
is Altered in Niemann-Pick Type C Disease (NPC) Model Cells”. Mol. Cell. Proteom..

[62] de
Araujo M. E. G., Liebscher G., Hess M. W., Huber L. A. (2020). Lysosomal
size matters. Traffic.

[63] Di
Lorenzo G., Velho R. V., Winter D., Thelen M., Ahmadi S., Schweizer M., De Pace R., Cornils K., Yorgan T. A., Grüb S. (2018). “Lysosomal Proteome
and Secretome Analysis Identifies Missorted Enzymes and Their Nondegraded
Substrates in Mucolipidosis III Mouse Cells”. Mol. Cell. Proteom..

[64] Brunk U. T., Dricsson J. L. (1972). Cytochemical evidence
for the leakage of acid phosphatase
through ultrastructurally intact lysosomal membranes. Histochem. J..

[65] Wang F., Gómez-Sintes R., Boya P. (2018). Lysosomal membrane permeabilization
and cell death. Traffic.

[66] Prentzell M. T. (2021). G3BPs tether the TSC
complex to lysosomes and suppress mTORC1 signaling. Cell.

[67] Ratto E., Chowdhury S. R., Siefert N. S., Schneider M., Wittmann M., Helm D., Palm W. (2022). Direct control of lysosomal
catabolic activity by mTORC1 through regulation of V-ATPase assembly. Nature Commun..

[68] Thelen M., Winter D., Braulke T., Gieselmann V. (2017). SILAC-Based
Comparative Proteomic Analysis of Lysosomes from Mammalian Cells Using
LC-MS/MS. Methods Mol. Biol..

[69] Schwake M., Schröder B., Saftig P. (2013). Lysosomal membrane proteins and their
central role in physiology. Traffic.

[70] Bové J., Martínez-Vicente M., Dehay B., Perier C., Recasens A., Bombrun A., Antonsson B., Vila M. (2014). BAX channel activity mediates lysosomal
disruption linked to Parkinson
disease. Autophagy.

[71] Dehay B., Bové J., Rodríguez-Muela N., Perier C., Recasens A., Boya P., Vila M. (2010). Pathogenic lysosomal
depletion in Parkinson’s disease. J.
Neurosci..

[72] Napolitano G., Johnson J. L., He J., Rocca C. J., Monfregola J., Pestonjamasp K., Cherqui S., Catz S. D. (2015). Impairment of chaperone-mediated
autophagy leads to selective lysosomal degradation defects in the
lysosomal storage disease cystinosis. EMBO Mol.
Med..

[73] Uzhytchak M., Smolková B., Lunova M., Jirsa M., Frtús A., Kubinová Š., Dejneka A., Lunov O. (2020). Iron Oxide
Nanoparticle-Induced Autophagic Flux Is Regulated by Interplay between
p53-mTOR Axis and Bcl-2 Signaling in Hepatic Cells. Cells.

[74] Arbab A. S., Wilson L. B., Ashari P., Jordan E. K., Lewis B. K., Frank J. A. (2005). A model of lysosomal metabolism of dextran coated superparamagnetic
iron oxide (SPIO) nanoparticles: implications for cellular magnetic
resonance imaging”. NMR In Biomedicine.

[75] Tassa C., Shaw S. Y., Weissleder R. (2011). Dextran-coated
iron oxide nanoparticles:
a versatile platform for targeted molecular imaging, molecular diagnostics,
and therapy. Acc. Chem. Res..

[76] Wu H. Y., Chung M. C., Wang C. C., Huang C. H., Liang H. J., Jan T. R. (2013). Iron oxide nanoparticles
suppress the production of
IL-1beta via the secretory lysosomal pathway in murine microglial
cells. Part Fibre Toxicol..

[77] Qasem R. J., Fallon J. K., Nautiyal M., Mosedale M., Smith P. C. (2021). Differential
Detergent Fractionation of Membrane Protein From Small Samples of
Hepatocytes and Liver Tissue for Quantitative Proteomic Analysis of
Drug Metabolizing Enzymes and Transporters. J. Pharm. Sci..

[78] Mahendran T., Kuznyetsova A., Moradi N., Hood D. A. (2025). Isolation
of functional
lysosomes from skeletal muscle. Am. J. Physiol.
Cell Physiol..

[79] Chen C., Sidransky E., Chen Y. (2022). Lyso-IP: Uncovering
Pathogenic Mechanisms
of Lysosomal Dysfunction. Biomolecules.

[80] Abu-Remaileh, M. ; Wyant, G. A. ; Kim, C. ; Laqtom, N. N. ; Abbasi, M. ; Chan, S. H. ; Freinkman, E. ; Sabatini, D. M. Lysosomal metabolomics reveals V-ATPase- and mTOR-dependent regulation of amino acid efflux from lysosomes. 2017, 358(6364), 807-813. 10.1126/science.aan6298. PMC5704967 29074583

[81] Liu Z., Lv Y. J., Song Y. P., Li X. H., Du Y. N., Wang C. H., Hu L. K. (2012). Lysosomal membrane protein TMEM192
deficiency triggers crosstalk between autophagy and apoptosis in HepG2
hepatoma cells. Oncol. Rep..

[82] Schröder B., Wrocklage C., Hasilik A., Saftig P. (2010). Molecular characterisation
of ‘transmembrane protein 192’ (TMEM192), a novel protein
of the lysosomal membrane”. Biol. Chem..

[83] Xiong, J. ; He, J. ; Xie, W. P. ; Hinojosa, E. ; Ambati, C. S. R. ; Putluri, N. ; Kim, H. E. ; Zhu, M. X. ; Du, G. Rapid affinity purification of intracellular organelles using a twin strep tag. J. Cell Sci.. 2019, 132(24). jcs235390 10.1242/jcs.235390. 31780580 PMC6955222

[84] Bonini S., Winter D. (2024). Two-Step Enrichment Facilitates Background Reduction
for Proteomic Analysis of Lysosomes. J. Proteome
Res..

