# Supplementary material for: Benchmarking Lysosome Enrichment Methods: A Guide for Research and Clinical Translation
Source: Anal Chem. 2026 Feb 3;98(6):4585–600. doi: 10.1021/acs.analchem.5c05792 (PMC12921670; doi:10.1021/acs.analchem.5c05792)
Supplement: Supplementary file 1 [file ac5c05792_si_001.pdf]

Supporting Information

Benchmarking lysosome enrichment methods: A guide for research and clinical translation

Anniek L de Jager[1], Sara Kassem[1], Alesha Louis[1], Brigitta AE Naber[1], Inge F de Laat[1], Bas de Mooij[1], Kyra van der Pan[1], Erik Bos[2], Roman I Koning[2], Jacques JM van Dongen[1, 3, *], Cristina Teodosio[1, 3, #, *], Paula Díez[1, 4, #, *]

[1] Department of Immunology, Leiden University Medical Center (LUMC), 2333ZA, Leiden, The Netherlands

[2] Electron Microscopy Facility, Department of Cell and Chemical Biology, Leiden University Medical Center (LUMC), 2333ZA, Leiden, The Netherlands

[3] Translational and Clinical Research Program, Cancer Research Center (IBMCC; University of Salamanca - CSIC); Cytometry Service, NUCLEUS; Department of Medicine, University of Salamanca and Institute of Biomedical Research of Salamanca (IBSAL), 37007, Salamanca, Spain

[4] Department of Functional Biology (immunology area), Faculty of Medicine and Health Sciences, University of Oviedo (UNIOVI), 33006, Oviedo, Spain

[#] C. Teodosio and P. Díez contributed equally to this manuscript

*** Corresponding authors**

- general topics: j.j.m.vandongen@eslho.org,
- proteomics studies: diezpaula@uniovi.es
- flow cytometric studies: cristina.teodosio@usal.es

Table of contents

Figure S1. Spectral flow cytometry data analysis workflow S2
Figure S2. Western blot images of selected organelle-specific proteins S3
Figure S3. Decision tree for selection of optimal lysosome enrichment method S4

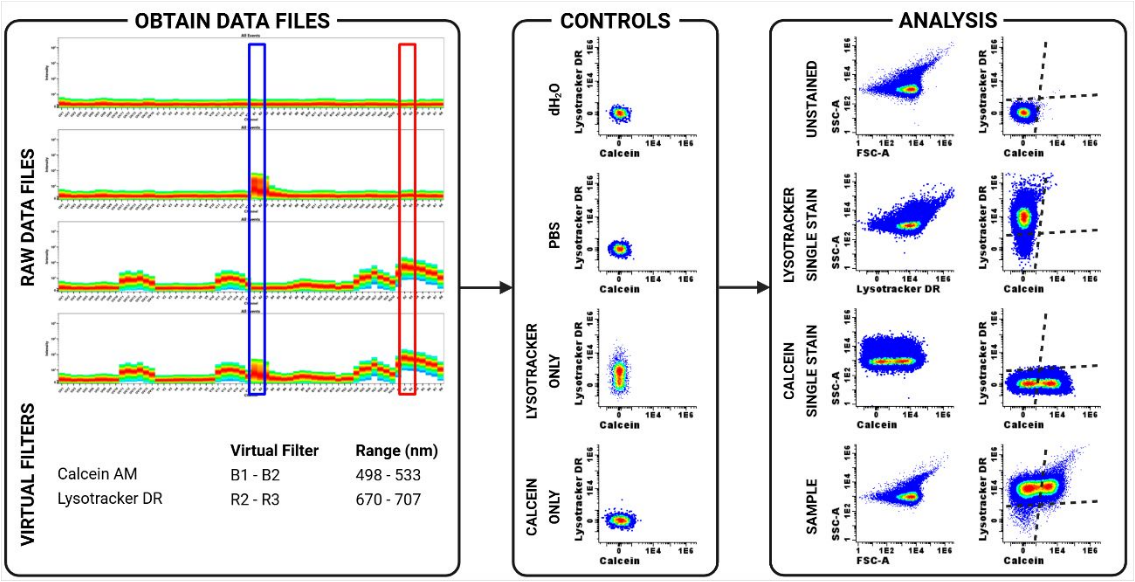

Figure S1. Spectral flow cytometry data analysis workflow. First, the obtained raw data files must be analysed using virtual filters as depicted in **A**. Here, the blue and red shapes display the virtual filter range used for Calcein and Lysotracker Deep Red, respectively. **B** shows the relevant controls measured to ensure minimal background and reagent contamination. Panel **C** displays the sample analysis, where the dotted lines show the location of the placed gates. *Lysotracker DR, Lysotracker Deep Red; dH$_2$O, distilled H$_2$O; PBS, phosphate-buffered saline.*

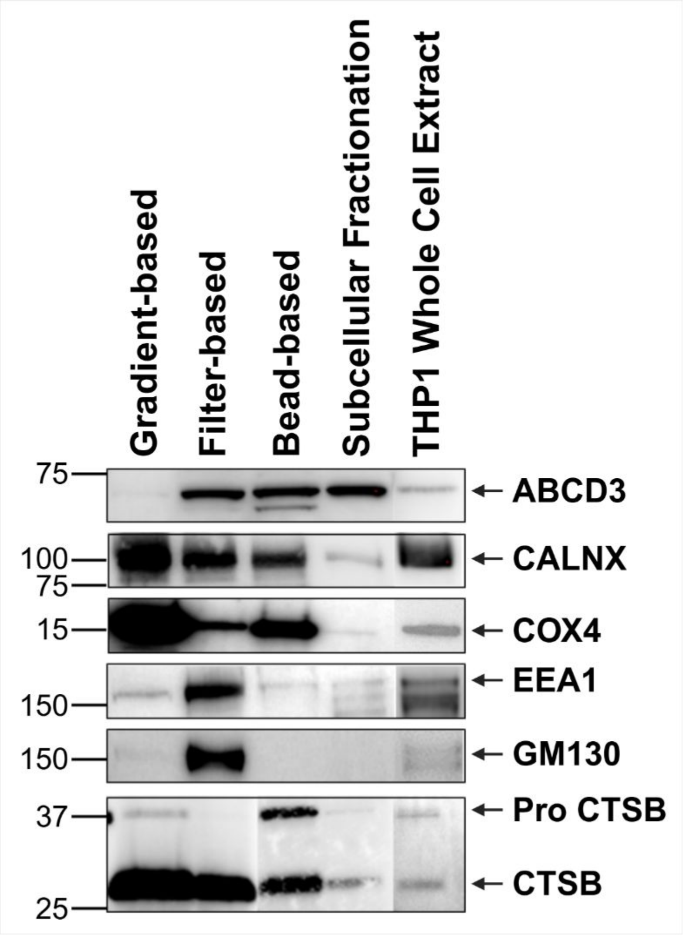

Figure S2. Western blot images of selected organelle-specific proteins. For western blotting, 10 µg of protein was loaded for COX4, GM130 and CLNX, and 20 µg of protein for EEA1, ABCD3 and CTSB.

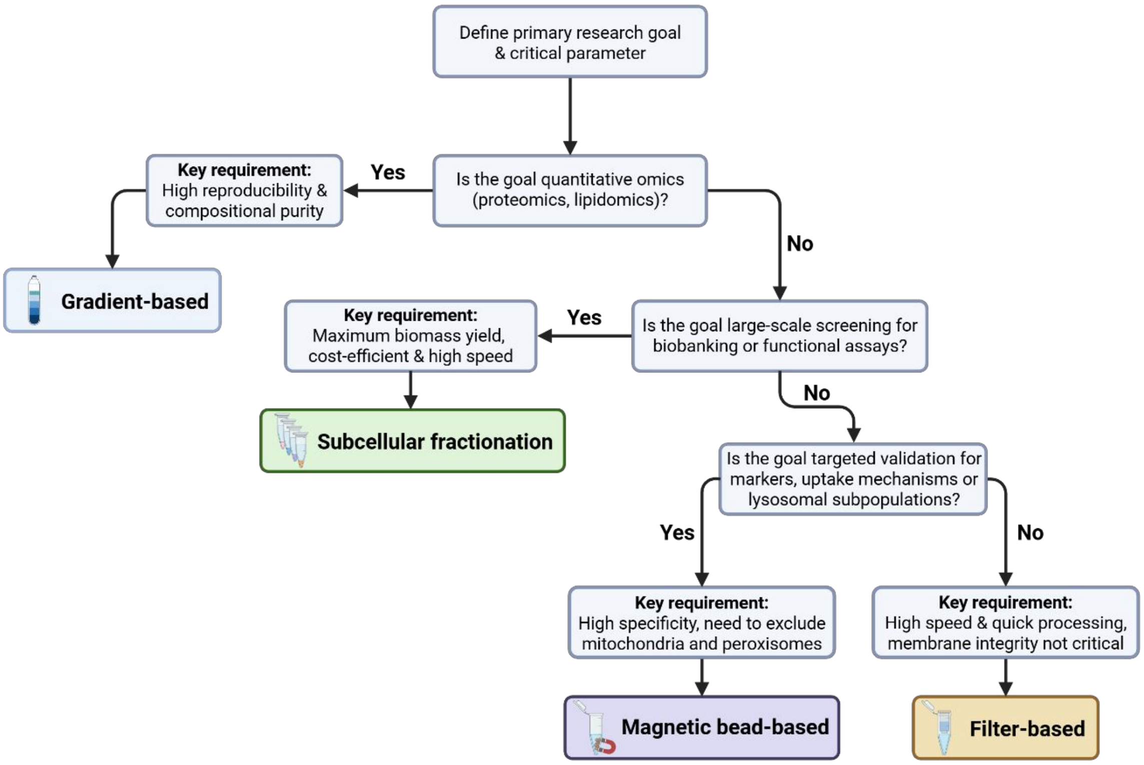

Figure S3. Decision tree for selection of optimal lysosome enrichment method. Created in https://BioRender.com
